# Membrane Potential Dynamics of Spontaneous and Visually Evoked Gamma Activity in V1 of Awake Mice

**DOI:** 10.1371/journal.pbio.1002383

**Published:** 2016-02-18

**Authors:** Quentin Perrenoud, Cyriel M. A. Pennartz, Luc J. Gentet

**Affiliations:** 1 Swammerdam Institute for Life Sciences, Center for Neuroscience, Faculty of Science, University of Amsterdam, the Netherlands; 2 Department of Neurobiology, Yale University School of Medicine, New Haven, Connecticut, United States of America; 3 Research Priority Program Brain and Cognition, University of Amsterdam, Amsterdam, the Netherlands; 4 Team Waking, Lyon Neuroscience Research Center, INSERM U1028 – CNRS UMR5292 F-69008, Lyon, France; 5 University Lyon 1, F-69000, Lyon, France; ICM - Institut du Cerveau et de la Moelle épinière Hôpital Pitié-Salpêtrière 47, bd de l'Hôpital, FRANCE

## Abstract

Cortical gamma activity (30–80 Hz) is believed to play important functions in neural computation and arises from the interplay of parvalbumin-expressing interneurons (PV) and pyramidal cells (PYRs). However, the subthreshold dynamics underlying its emergence in the cortex of awake animals remain unclear. Here, we characterized the intracellular dynamics of PVs and PYRs during spontaneous and visually evoked gamma activity in layers 2/3 of V1 of awake mice using targeted patch-clamp recordings and synchronous local field potentials (LFPs). Strong gamma activity patterned in short bouts (one to three cycles), occurred when PVs and PYRs were depolarizing and entrained their membrane potential dynamics regardless of the presence of visual stimulation. PV firing phase locked unconditionally to gamma activity. However, PYRs only phase locked to visually evoked gamma bouts. Taken together, our results indicate that gamma activity corresponds to short pulses of correlated background synaptic activity synchronizing the output of cortical neurons depending on external sensory drive.

## Abstract 2

Gamma activity, an important component of brain dynamics, is driven by synaptic background activity and synchronizes distinct cortical cell types differently depending on visual input.

## Author Summary

The neocortex is the main substrate of cognitive activity of the mammalian brain. During active wakefulness, it exhibits an oscillatory activity in the gamma range (30–80Hz), which is believed to play an important functional role and is altered in schizophrenic patients. Experimental studies have shown that gamma activity arises from the interaction of excitatory pyramidal neurons, the main neuronal type of the cortex, and local inhibitory neurons expressing the protein parvalbumin (PV). However, how these neuronal types behave during gamma activity remains largely unknown. Here, we recorded the intracellular activity of pyramidal and PV-expressing neurons in the visual cortex of awake mice while acquiring Local Field Potentials (LFPs)—extracellular voltage fluctuations within a small volume of the cortex—to monitor gamma activity. We found that gamma activity arises when PV-expressing neurons synchronize their output in response to a correlated input, reflecting the general activation of the local cortical network. This happens even in the absence of visual input. On the other hand, the output of pyramidal neurons only becomes entrained to gamma activity when the mice are exposed to visual stimulation. Thus, our results suggest that gamma activity synchronizes pyramidal neurons specifically when the cortex is engaged in processing external inputs.

## Introduction

Cortical activity in the gamma range (30–80 Hz) has been the focus of considerable attention in the last two decades. Gamma activity is impaired in schizophrenic patients [1] and has been hypothesized to play an important role in attention, inter-areal communication, and the synchronization of local activity [2–4]. However, the function of gamma activity is still debated [5,6]. In addition, while several functional models have been proposed, how gamma activity directly relates to the dynamics of the gamma rhythmogenic network during awake sensory processing has never been observed [4,7]. As a result, our knowledge of the potential constraints applying to a realistic theoretical description of gamma activity remains incomplete.

Gamma band activity has been primarily studied using extracellular electrodes in the visual cortex of cats and monkeys [8,9], where it is evoked in the Local Field Potential (LFP) by visual stimuli such as drifting gratings. Gamma phase locking of extracellularly recorded units is most prominent in layers 2/3 [10], increases with selective attention [8], and correlates with shortened reaction times as well as maximized signal to noise ratios [11,12]. This synchronization is believed to improve local processing and to facilitate the transfer of information to higher-order cortical areas [4,7,13–18]. Recent studies using optogenetics in rodents have brought support to this hypothesis, showing that gamma activity improves tactile detection [19] and depends on the activity of parvalbumin-expressing fast-spiking interneurons (PV) [20,21].

Numerous computational studies have described how gamma rhythmicity can arise from networks of PVs and pyramidal excitatory neurons (PYRs) [22,23]. However, these models typically describe how oscillatory activity emerges under stereotypical steady-state regimes [24]. By contrast, it has been recently shown that in vivo gamma activity is an unstructured phenomenon that patterns temporally in a way that is similar to filtered white noise [25,26]. This illustrates that cortical gamma activity may not be an oscillation per se, but a stochastic process containing transient bouts of activity having energy in the gamma range [19]. The dynamics linking gamma rhythmicity to the subthreshold activity of PVs and PYRs have only been studied in brain slices using pharmacological manipulation [23,27,28] or in anesthetized animals, where brain activity is characterized by a stereotypical alternation of hyperpolarized and depolarized states [29–31] and where visual processing and GABAergic inhibition are strongly affected [32,33]. Thus the experimental data required to constrain a realistic model of the temporal patterning of gamma activity and of the way it entrains cortical neurons under naturalistic awake conditions are still missing.

To address this issue, we characterized the intracellular correlates of spontaneous and visually evoked gamma rhythmicity in PVs and PYRs of layers 2/3 in V1 of awake mice by performing whole-cell recordings synchronously with nearby LFP recordings. Gamma power in LFPs was correlated to the depolarization of the membrane potential (Vm) of PVs and PYRs on a coarse and fine time scale, indicating that gamma activity is expressed in response to the background synaptic input underlying their subthreshold dynamics. Strong bouts of LFP gamma activity rarely persisted for more than one to three cycles, occurring more frequently during visual stimulation but also occurred spontaneously. While the firing of PVs was entrained by spontaneous and visually evoked gamma bouts, the firing of PYRs only phase locked to gamma during visual stimulation. Taken together, our findings indicate that gamma activity emerges in response to correlated background synaptic activity and that layers 2/3 pyramidal neurons synchronize their firing to gamma activity selectively when engaged in visual processing.

## Results

**Fig 1:**
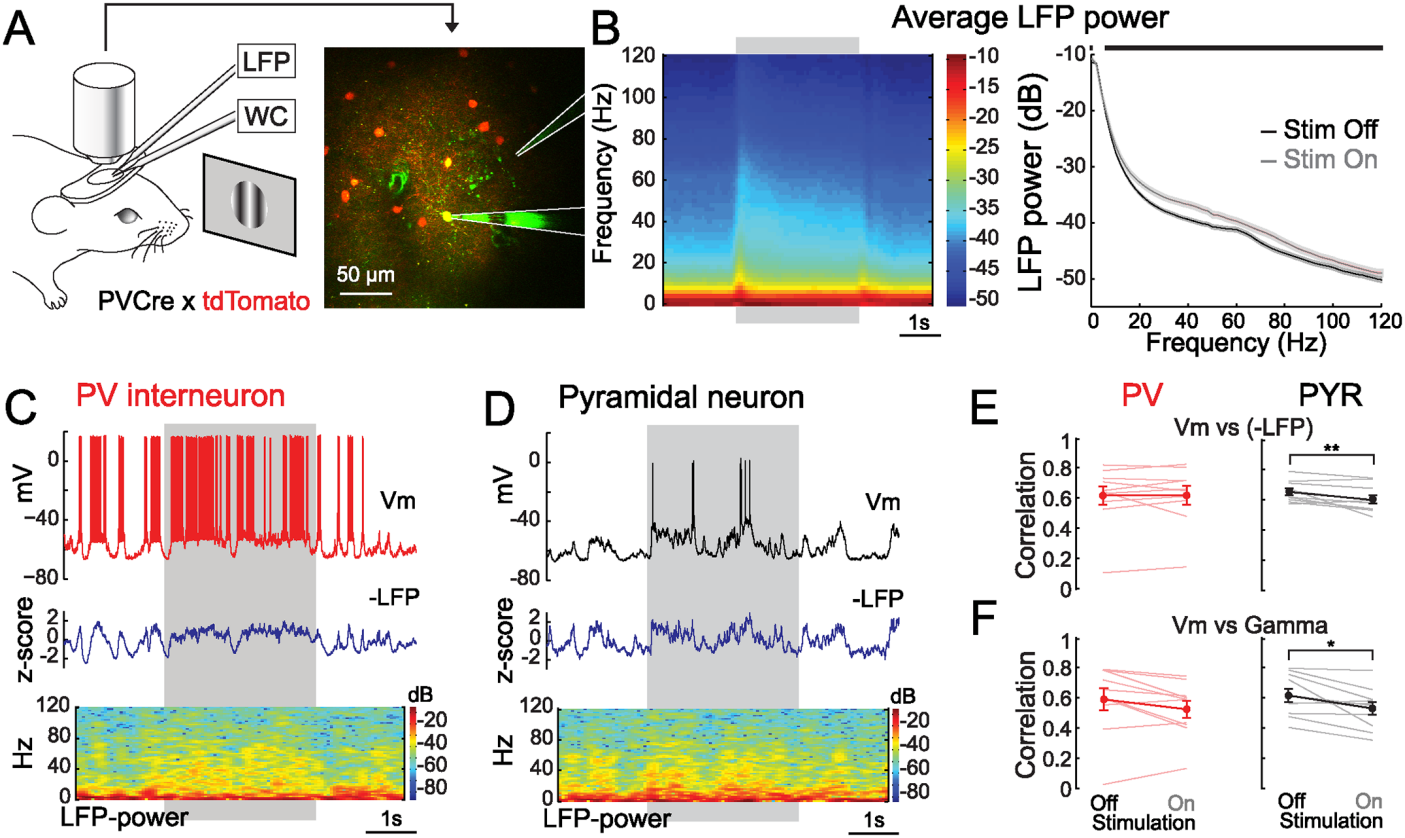
LFP Gamma power correlates with the membrane potential dynamics of PVs and PYRs. (**A**) Experimental design: LFP and two-photon targeted whole-cell (WC) or cell attached recordings of V1 L2/3 PVs and PYRs are performed in awake mice visually stimulated with drifting gratings. Right: micrograph taken during an example PV whole-cell recording. (**B**) Visual stimulation elicits an average increase in LFP power in the beta (12–28 Hz) and gamma (30–80 Hz) range. Left: grand mean spectro-temporal representation of LFP power around stimulation (*n* = 34; grey rectangle: visual stimulation period). Right: average power spectra during (Stim On: grey) and outside (Stim off: black) visual stimulation (shaded areas: +/- standard error of the mean (s.e.m); horizontal black line: statistical significance, False Detection Rate (FDR) corrected signed-rank test, α = 0.05). (**C, D**) Simultaneous LFP and whole-cell recordings of a PV (C) and a PYR (D) (Top: whole-cell recording; Middle: inverted LFP recording; Bottom: spectro-temporal representation of the LFP; grey rectangle: visual stimulation period). (**E**) Vm is correlated with the inverted LFP (-LFP) in PVs (*n* = 10) and PYR (*n* = 10; thin lines: individual neurons; thick line and filled circles with error bars: mean +/- s.e.m; **: *p* < 0.01, signed-rank test). (**F**) Vm is correlated with gamma power in PVs (*n* = 10) and PYRs (*n* = 10; thin lines: individual neurons; thick line and filled circles with error bars: mean +/- s.e.m; *: *p* < 0.05, signed-rank test).

To determine the intracellular correlates of gamma activity in V1, we performed two-photon targeted patch-clamp (TPTP) recordings of pyramidal cells (PYRs) and parvalbumin-expressing interneurons (PVs) combined with local field potential (LFP) recordings in layers 2/3 of head-fixed awake mice (Fig 1A; Materials and Methods). Recordings were obtained in the whole-cell (PYR: *n* = 10; PV: *n* = 10) or cell-attached configuration (PYR: *n* = 1; PV: *n* = 13). One whole-cell PYR recording did not yield spike and was only used for the analysis of membrane potentials (Vm). LFPs were acquired simultaneously with glass pipettes positioned close to the recorded cell (Distance (μm); PYR: min: 76.6, max: 301.5, median: 200.2; PV: min: 22.9, max: 311.1, median: 155.3). Inter-channel distances were not significantly different between PYR and PV recordings (ranksum, *p* = 0.16). As pipette resistance can affect the amplitude of small signals, LFP recordings were normalized and expressed as z-scores (Fig 1C and 1D). Drifting gratings were displayed at eight different orientations on a screen placed in front of the eye of the animal contralateral to the recorded hemisphere (Stim On). A grey isoluminant background was displayed between epochs of visual stimulation (Stim Off, Materials and Methods).

All PV interneurons displayed fast spikes (Peak-to-trough time: 1.2 ± 0.2 ms; *n* = 10) and high firing rates consistent with former reports [34–36] (Stim Off: 33.9 ± 4.5 Hz; Stim On: 55.1 ± 6.1 Hz; *p* < 0.001; *n* = 23). No significant difference was found between the firing rates of PVs recorded in the whole-cell (*n* = 10) or the cell-attached (*n* = 13) configuration (ranksum, Stim Off: *p* = 0.83; Stim On: *p* = 0.93). PYR cells had slower spikes (Peak-to-trough time: 6.5 ± 0.4 ms; *n* = 9) and lower firing rates (Stim Off: 1.2 ± 0.4 Hz; Stim On: 1.7 ± 0.8 Hz, *p* < 0.05; *n* = 10) as reported previously [35]. In accordance with previous studies [37–39], we found that pyramidal neurons responded selectively to particular grating orientations (Orientation Selectivity Index [OSI]: 0.54 ± 0.08; *n* = 10) while PV cells were generally either broadly orientation-tuned or untuned (OSI: 0.15 ± 0.02; *n* = 22). LFP power decreased with frequency and presented a shoulder around 60 Hz in the absence of visual input while visual stimulation resulted in a broadband increase in average LFP power (Materials and Methods) in the beta (12–28 Hz) and gamma (30–80 Hz) ranges (Fig 1B) matching previous observations in mouse V1 [40]. No significant difference was observed at any frequency between the LFP spectra recorded simultaneously with PYR cells and those recorded with PV interneurons (Stim Off: lowest *p*-value: 0.17 at 2 Hz; Stim On: lowest *p*-value: 0.11 at 2 Hz).

### Gamma Power Correlates with the Membrane Potential Dynamics of Pyramidal and PV Neurons

We next inspected how Vm, LFP and LFP power spectra varied over time in individuals trials (Fig 1C and 1D). LFP signals recorded in layer 2/3 are a reflection of the background synaptic activity shared by pyramidal cells and PV interneurons, which entrains their membrane potential dynamics [41–45]. Accordingly, we found that the inverse of the LFP was highly correlated to the membrane potentials of both PYRs and PVs (Fig 1E). Interestingly, epochs of strong gamma activity were concurrent with Vm depolarization in both PYRs and PVs (Fig 1C and 1D). To quantify this phenomenon, we computed the correlation between LFP power in the gamma range (30–80 Hz) and mean Vm in non-overlapping 500 ms windows. Gamma power was strongly correlated with membrane depolarization during Stim Off and Stim On periods in both types of neurons (Fig 1F). Accordingly, linear regressions indicated that Vm explained a noticeable fraction of the variance of gamma power (Explained Variance: PV: Stim Off: 40.9 ± 6.7%; Stim On: 31.4 ± 5.4%; n = 10; PYR: Stim Off: 41.0 ± 5.6%; Stim On: 30.8 ± 4.9%; *n* = 10). Thus, our experiments indicate that LFP gamma activity occurs when PYRs and PVs depolarize in response to correlated background synaptic inputs.

### Strong Gamma Rhythmicity Occurs in Short Bouts

**Fig 2:**
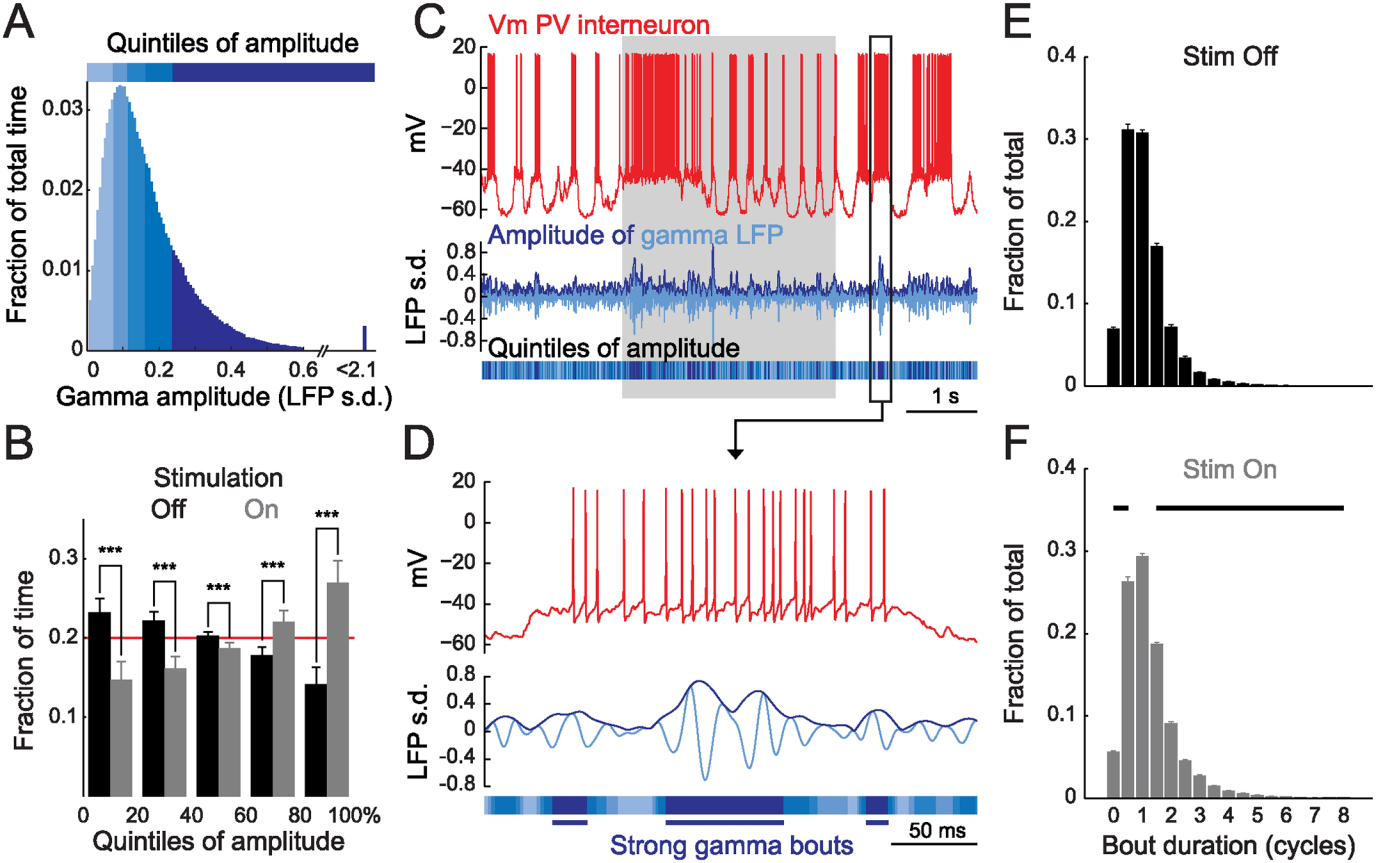
Gamma occurs in short bouts. (**A**) Distribution of LFP gamma amplitude over time in an example PV recording. Quintiles are color-coded from light to dark blue as a function of gamma amplitude and represent amplitude ranges occurring during one-fifth of the recording time. (**B**) Visual stimulation biases LFP gamma amplitude toward the range of strong gamma quintiles (*n* = 34; red line: overall fraction of time spent in each quintile; error bars: s.e.m.; ***: *p* < 0.001, signed-rank test). (**C**) Example trace of the recording shown in (A) (top: whole-cell recording; middle: gamma-filtered LFP (light blue) and gamma amplitude envelope computed with the Hilbert transform (dark blue); bottom: gamma quintiles color coded as in (A); grey rectangle: visual stimulation period). (**D**) Enlargement of the portion enclosed in the black rectangle in (C) showing examples of spontaneous gamma bouts. (**E, F**) Distribution of the duration of gamma bouts outside (E, Stim Off) and during (F, Stim On) visual stimulation in gamma cycles (number hemicycles of the gamma filtered LFP divided by two; *n* = 34; error bars: s.e.m.; black line in (F): statistical difference between Stim Off and Stim On, FDR-corrected signed-rank test, α = 0.05).

To analyze the dynamics of gamma activity on a finer time scale and in terms of both amplitude and phase, we computed the instantaneous amplitude of gamma using the Hilbert transform of the 30–80 Hz filtered LFP and divided its distribution over time in five quintiles (Fig 2A; Materials and Methods). Gamma amplitude remained weak in the four lower quintiles (range: 0 to 0.19 +/- 0.05 LFP s.d.) while it spanned a much wider range in the strongest gamma quintile (maximum amplitude: 1.4 +/- 0.8 LFP s.d.; *n* = 34). Visual stimulation resulted in a significant increase of the total time spent in the two strongest gamma quintile and a significant decrease of the total time spent in the three weakest (Fig 2B) consistent with the increased average LFP gamma power observed during epochs of visual activity.

Examining how gamma amplitude fluctuated in individual trials (Fig 2C and 2D), we found that gamma oscillations only remained in the range of the strongest quintile during short periods typically encompassing one to three cycles (Fig 2D–2F, Materials and Methods). Visual stimulation resulted in a slight but significant increase in the average duration of strong gamma events (epoch duration: Stim Off: 20.4 ± 1.1 ms; Stim On: 24.0 ± 3.2, *p* < 0.001, *n* = 34). However the duration of spontaneous and visually evoked strong gamma events remained in the same range (Fig 2E and 2F). Thus, as recently observed [19,25,26], our results indicate that gamma activity is not per se an oscillation but rather occurs in short synchronizing bouts.

### The Firing of PV Interneurons Is Phase Locked to Strong Gamma Oscillations

**Fig 3:**
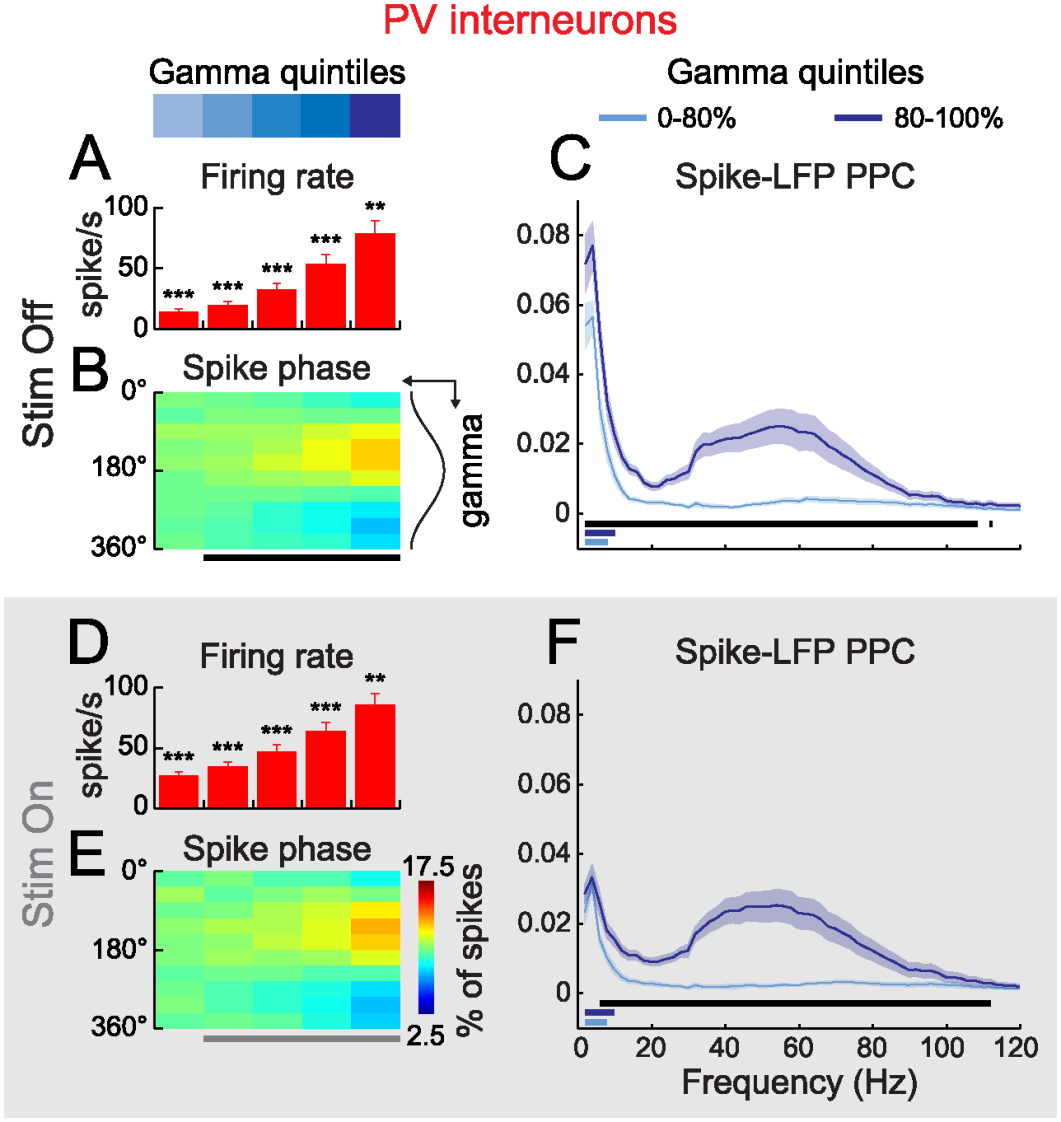
The firing of PVs phase locks to strong gamma oscillations. (**A, D**) Grand mean firing rate of PVs, outside (A, Stim Off) and during (D, Stim On) visual stimulation, as a function of gamma quintile at spike time (*n* = 23; error bars: s.e.m.; **: *p* < 0.01, ***: *p* < 0.001, signed rank test). (**B, E**) PV spikes occur preferentially before the trough of strong gamma outside (B) and during (E) visual stimulation (color-code in (E): fraction of spikes falling in one of ten bins of gamma phase as a function of gamma quintile at spike time; black and gray lines in (B) and (E): statistical difference from a uniform distribution, Rayleigh’s test, α = 10^−5^). (**C, F**) Strong gamma increases Spike-LFP Pairwise Phase Consistency (PPC) in the gamma range (30–80 Hz) outside (C) and during (F) visual stimulation (*n* = 23; light and dark blue traces: grand mean PPC respectively in the four weakest quintiles and in the strongest gamma quintile; shaded areas: +/- s.e.m; horizontal lines: statistical significance between the four weakest quintiles and the strongest gamma quintile (black) and between Stim Off and Stim On for the four weakest quintiles (light blue) and the strongest gamma quintile (dark blue), FDR corrected signed-rank test, α = 0.05).

Gamma rhythmicity has been linked to the activity of PV cells [20,21,30]. To investigate this link, we sorted PV spikes per gamma quintile during and in the absence of visual stimulation. The average firing rate of PV cells increased gradually in successive quintiles, indicating that LFP gamma is associated with a higher probability of firing (Fig 3A and 3D). We then computed the phase of gamma-filtered LFP at spike time and assigned spikes to 10 phase bins in each quintile. This revealed a significant accumulation of spikes immediately preceding gamma troughs as gamma strength increased (Fig 3B and 3E). Thus, strong gamma events seem to synchronize PVs firing.

To examine the temporal structure of gamma spike phase locking in PVs in more detail, we computed the autocorrelograms of PVs firing and the spike-triggered averages (STAs) of the gamma-filtered LFP as a function of gamma quintile (S1 Fig). STAs displayed an increasing oscillatory tendency in successive quintiles (S1B and S1D Fig), confirming that the spikes of PV cells occur at a preferential phase during strong gamma bouts. Interestingly however, spike autocorrelograms were similar between quintiles and did not show rhythmicity in the gamma range (S1A and S1C Fig). Similarly, action potential threshold was unchanged between gamma quintiles (S1F Fig). Thus our results suggest that strong gamma activity does not correlate with a stereotypical firing rhythmicity at the level of single PV neurons, but rather that gamma synchronizes their collective activity on a short time scale.

Notably, we found little difference in the result of these analyses between baseline and visual stimulation. Visual stimulation resulted in an overall increase in neuronal firing rates (Fig 3A and 3D; Stim Off: 33.9 ± 4.5 Hz; Stim On: 55.1 ± 6.1 Hz; *p* < 0.001; *n* = 23) and a moderate but significant increase in the power of STAs in the strongest gamma quintile (S1E Fig). Stimulation might however affect the preferred frequency of gamma phase locking. In order to examine this we estimated phase locking over a broad range of LFP frequencies (2–120 Hz) using the Pairwise Phase Consistency (PPC, Materials and Methods). PPC is an unbiased measure of spike-LFP phase locking and has an expected value of zero if spikes are uniformly distributed over phases. Phase locking spectra were similar between baseline and stimulation in the gamma range (Fig 3C and 3F). During and outside visual stimulation, PPC was close to zero in the four weakest gamma quintiles and displayed a significant and broadly distributed increase during strong bouts (80%–100% quintile; Fig 3C and 3F). Thus, our data indicate that the mechanisms linking gamma rhythmicity to the firing of PV cells are similar during baseline activity and visual stimulation. In both cases, PV firing was tightly coupled to strong gamma bouts.

PPC estimates can be unreliable if spike samples are not large enough (Materials and Methods). To make sure that our PPC results were not an artifact resulting from variations of spike count, we recomputed our analysis on sets containing a fixed number of randomly resampled spikes (125, 250, 500, and 1,000 spikes; S2 Fig). Spikes were resampled without repetition and this operation was performed 1,000 times per condition and per neuron. The variability of PPC decreased as spike count increased and the estimates converged to our raw PPC estimates for spike counts of 500 and 1,000 spikes. This indicates that the increased gamma phase locking PVs during strong gamma bouts cannot be accounted for by variability in spike sample size.

### Strong Gamma Oscillations Entrain Membrane Potential Fluctuations in PV Interneurons

**Fig 4:**
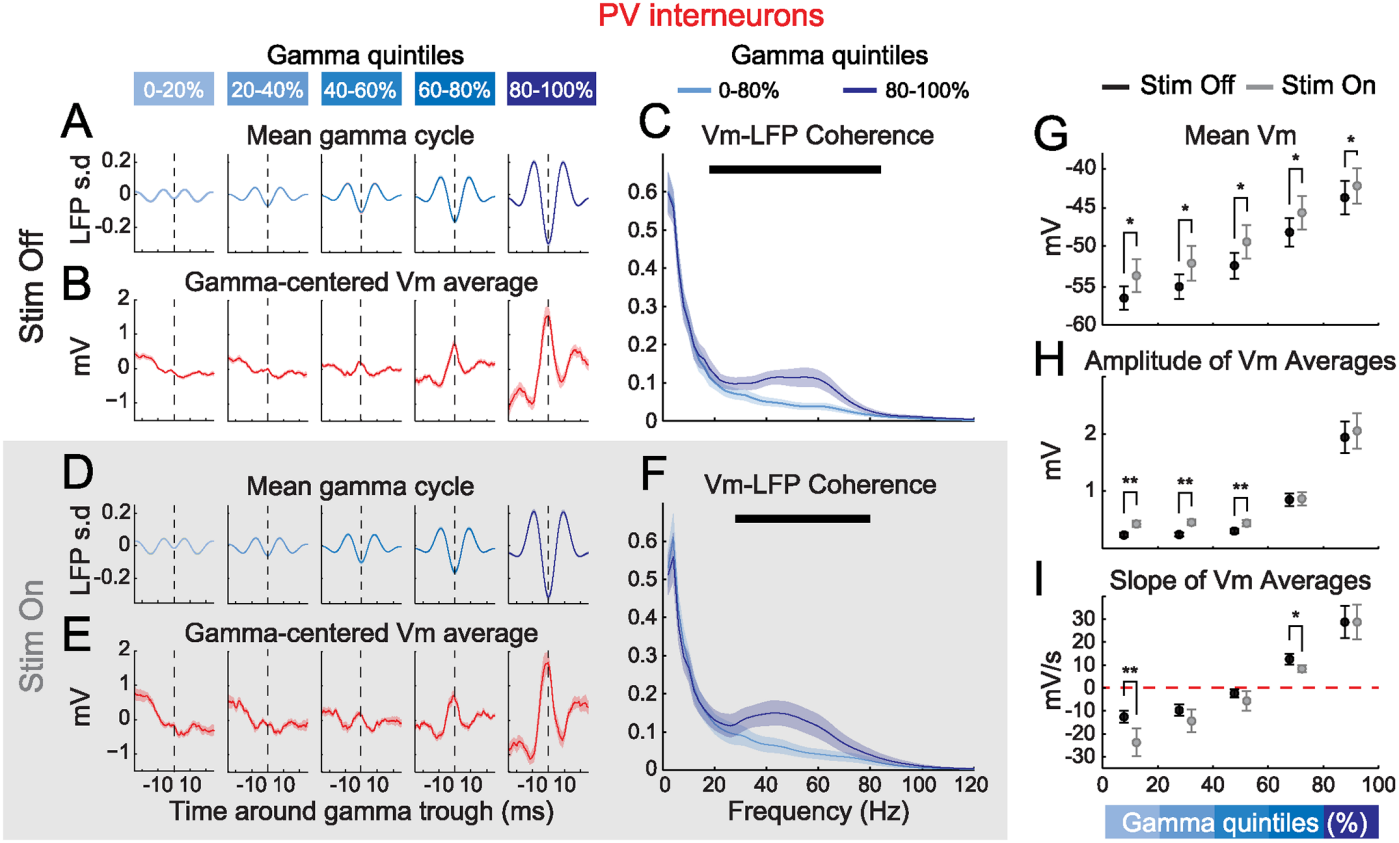
Strong gamma oscillations entrain Vm in PVs and occur when PVs depolarize. (**A, D**) Grand mean of trough-centered segments of the gamma-filtered LFP, outside (A) and during (D) visual stimulation, as a function of gamma quintile at trough time (*n* = 10; shaded areas: +/- s.e.m.). (**B, E**) Grand mean of simultaneously recorded PV Vm segments (*n* = 10; shaded areas: +/- s.e.m.). (**C, F**) Strong gamma oscillations increase PV Vm-LFP coherence in the gamma range (30–80 Hz) outside (C) and during (F) visual stimulation (*n* = 10; light and dark blue traces: grand mean coherence respectively in the four weakest quintiles and in the strongest gamma quintile; shaded areas: +/- s.e.m; black horizontal line: statistical significance between the four weakest quintiles and the strongest gamma quintile, FDR corrected signed-rank test, α = 0.05). (**G**) Grand mean DC Vm of PVs as a function of gamma quintile outside (Stim Off, black) and during (Stim On, grey) visual stimulation (*n* = 10; error bars: s.e.m.; *: *p* < 0.05, signed rank test). (**H**) Grand mean amplitude of gamma-centered Vm averages outside (Stim Off, black) and during (Stim On, grey) visual stimulation (*n* = 10; error bars: s.e.m.; **: *p* < 0.01, signed rank test). (**I**) Grand mean slope of linear fits to gamma-centered Vm averages outside (Stim Off, black) and during (Stim On, grey) visual stimulation (*n* = 10; error bars: s.e.m.; *: *p* < 0.05, **: *p* < 0.01, signed-rank test).

We next analyzed the relationship of the Vm of PV interneurons to gamma activity. Spikes were removed from Vm traces from 1 ms prior to peak to 3 ms post peak, and missing points were interpolated with cubic splines (Materials and Methods). Cycles of gamma-filtered LFPs recorded during or outside visual stimulation were grouped as a function of gamma quintile at trough time, aligned and averaged (Fig 4A and 4D). Synchronously recorded Vm segments were grouped and aligned similarly to produce averages of the membrane potential dynamics of PVs centered on gamma trough. These gamma-centered Vm averages displayed increased oscillatory behaviors in successive quintiles (Fig 4B, 4E and 4H). Thus, the synchronization of PVs during strong gamma bouts results from a transient and synchronous oscillation of their membrane potentials in the gamma range. Interestingly, gamma-centered Vm averages were depolarizing in high amplitude gamma quintiles and hyperpolarizing in weak quintiles. To quantify this phenomenon, we computed linear fits to each gamma-centered Vm average. The slopes of these fits were negative for the three weakest gamma quintiles and positive for the two strongest (Fig 4I). Therefore, our results indicate that strong gamma rhythmicity occurs when PV neurons are depolarizing.

While we found little difference in the slopes of these fits between Stim On and Stim Off epochs, visual stimulation resulted in a significant overall increase of the DC (direct current) level of depolarization of PV cells (Fig 4G), and a significant decrease in Vm slopes in the first four gamma quintiles (Fig 4I). In order to assess whether stimulation could affect the preferred frequency of gamma phase locking, we computed the coherence of the LFP and Vm over a wide range of frequencies (2–120 Hz) during strong gamma bouts and for the remaining quintiles (Fig 4C and 4F). We found no significant difference in Vm-LFP coherences between Stim Off and Stim On in the gamma range (30–80 Hz). In both cases, coherence remained low in weak gamma quintiles and increased significantly during strong gamma bouts (Fig 4C and 4F).

To make sure that the Vm dynamics that we observed here were not an artefact of spike removal, we reproduced our analyses on current clamps recordings of PVs performed while current was injected to maintain Vm under the threshold of spike initiation (S3 Fig; *n* = 6). The results of these analyses were highly similar to those on spike-removed traces. This indicates that the synchronization of Vm dynamics during strong gamma bouts in PV cannot be accounted for by the intrinsic active conductances underlying spike initiation and repolarization.

To further understand how the synchronization of the membrane potential dynamics of PVs arises during strong gamma bouts, we performed voltage clamp recordings on a subset of PVs (S4 Fig). PV neurons were held at either -80 mV or at +10 mV to bias transmembrane current toward excitatory (EPSCs) or inhibitory post synaptic currents (IPSCs) respectively. For both IPSCs and EPSCs, gamma phase locking tended to increase during strong gamma bouts in Stim On and Stim Off periods (S4K–S4N Fig). Average EPSCs, in particular, locked strongly to gamma cycles as gamma strength increased (S4D and S4H Fig), whereas putative IPSCs seemed to display a weaker relationship. Thus, our results suggest that, at least, excitatory currents play an important role in the entrainment of PVs to strong gamma bouts. It should be pointed out, however, that our data do not rule out an additional role of IPSCs in the recruitment of PV neurons. Indeed, holding potentials of +10 mV are accompanied by higher noise levels. Thus, possible contamination by other currents might mask some of the IPSCs phase locking.

### Strong Gamma Oscillations Entrain Spiking in Pyramidal Neurons during Visual Stimulation

**Fig 5:**
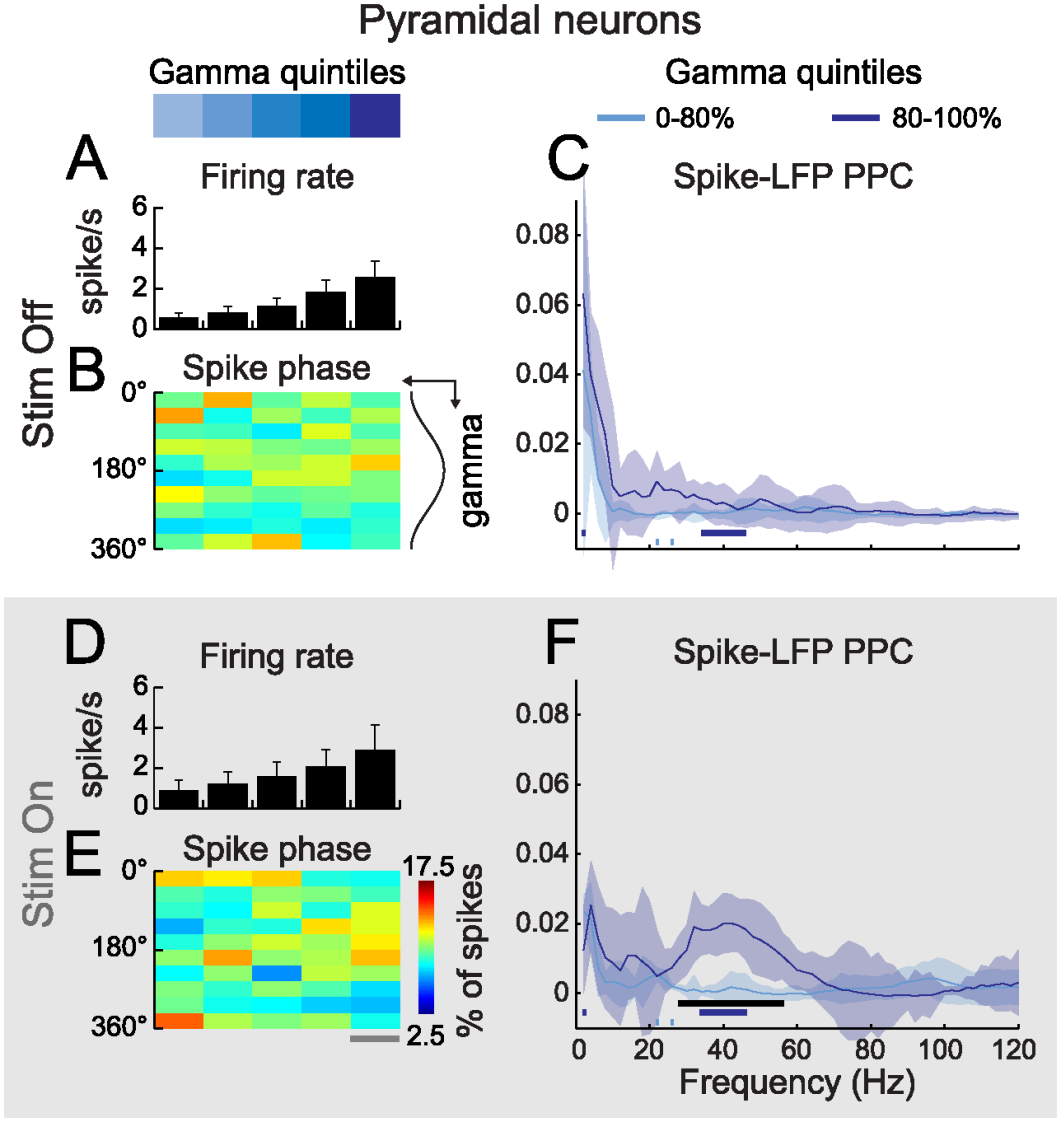
The firing of PYRs phase locks to strong gamma oscillations during visual stimulation. (**A, D**) Grand mean firing rate of PYRs, outside (A, Stim Off) and during (D, Stim On) visual stimulation, as a function of gamma quintile at spike time (*n* = 10; error bars: s.e.m.). (**B, E**) PYR spikes occur preferentially before the trough of strong gamma oscillations during (E) but not outside (B) visual stimulation (color-code in (E): fraction of spikes falling in one of ten bins of gamma phase as a function of gamma quintile at spike time; gray line in (E): statistical difference from a uniform distribution, Rayleigh’s test, α = 10^−5^). (**C, F**) Strong gamma increases spike-LFP PPC in the gamma range (30–80 Hz) during visual stimulation (F) but not outside (C) (*n* = 10; light and dark blue traces: pooled PPC respectively in the four weakest quintiles and in the strongest gamma quintile; shaded areas: +/- Jackknife 95% confidence interval; horizontal lines: statistical significance between the four weakest quintiles and the strongest gamma quintile [black] and between Stim Off and Stim On for the four weakest quintiles [light blue] and the strongest gamma quintile [dark blue], Materials and Methods).

We next asked whether gamma entrains the firing of layer 2/3 pyramidal neurons. As PYRs have low firing rates, we pooled spikes occurring in the first four quintiles of gamma amplitude into a single “weak gamma” group so as to gain statistical power for STAs and autocorrelograms (S5 Fig). During and outside visual stimulation, the average firing rates of PYRs increased in successive gamma quintiles (Fig 5A and 5D), while autocorrelograms remained unchanged (S5A and S5C Fig). Thus, as for PV cells, strong gamma activity does not appear to affect the temporal structure of spike trains at the level of single PYRs, but rather to correlate with a higher probability of firing. In the absence of visual stimulation, STAs displayed no or little oscillatory behaviors in weak LFP gamma quintiles and displayed only a weak modulation in the strongest quintile (S5B Fig). Accordingly, histograms of spike phase did not reveal a significant phase preference under this condition (Fig 5B). During visual stimulation however, while STAs only displayed a slight non-significant increase in oscillatory tendency in the strongest gamma quintile (S5D and S5E Fig), phase histograms revealed a significant accumulation of spikes before the gamma trough during strong gamma bouts (Fig 5E). To explore this further, PPC was computed for weak gamma and strong gamma over 2–120 Hz and compared between Stim Off and Stim On (Fig 5C and 5F). In both cases, PPC remained close to zero in the weak gamma group. During baseline, strong gamma did not correlate with significantly increased PPC in the gamma range (Fig 5C). However, strong gamma resulted in a significant increase in gamma phase locking during stimulation (Fig 5F). Thus, in contrast to PVs, our data indicate that gamma spike phase locking is modulated by visual stimulation in Pyramidal neurons. To confirm that this result was not an artifact resulting from variations in sample size between Stim Off and Stim On period, we recomputed PPCs on subsets of our pooled sample containing a fixed number of spikes (125, 250, 500, and 1,000 spikes; S6 Fig). Spikes were resampled without repetition and this operation was performed 1,000 times per condition. For counts of 1,000 spikes, estimates converged toward our raw PPC estimates. This indicates that Stim On specific gamma phase locking in PYRs cannot be accounted for by variations in spike sample size between baseline and visual stimulation periods.

### Strong Gamma Oscillations Entrain Membrane Potential Fluctuations in Pyramidal Neurons

**Fig 6:**
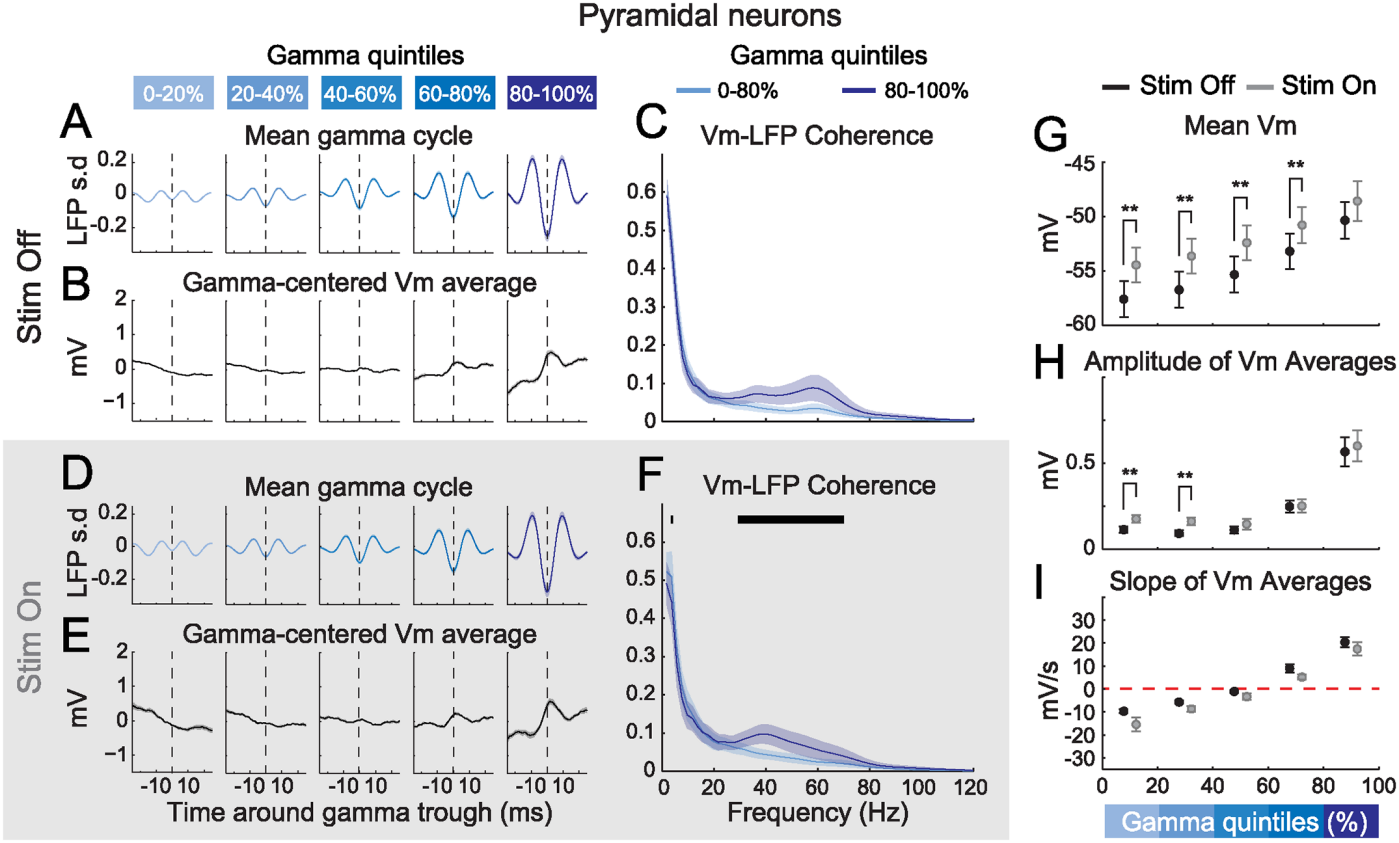
Strong gamma oscillations entrain Vm in PYRs and occur when PYRs depolarize. (**A, D**) Grand mean of trough-centered segments of the gamma-filtered LFP, outside (A) and during (D) visual stimulation, as a function of gamma quintile at trough time (*n* = 10; shaded areas: +/- s.e.m.). (**B, E**) Grand mean of simultaneously recorded PYR Vm segments (*n* = 10; shaded areas: +/- s.e.m.). (**C, F**) Strong gamma oscillations increase PYR Vm-LFP coherence in the gamma range (30–80 Hz) outside (C) and during (F) visual stimulation (*n* = 10; light and dark blue traces: grand mean coherence respectively in the four weakest quintiles and in the strongest gamma quintile; shaded areas: +/- s.e.m; black horizontal line: statistical significance between the four weakest quintiles and the strongest gamma quintile, FDR corrected signed-rank test, α = 0.05). (**G**) Grand mean DC Vm of PYRs as a function of gamma quintile outside (Stim Off, black) and during (Stim On, grey) visual stimulation (*n* = 10; error bars: s.e.m.; **: *p* < 0.01, signed-rank test). (**H**) Grand mean amplitude of gamma-centered Vm averages outside (Stim Off, black) and during (Stim On, grey) visual stimulation (*n* = 10; error bars: s.e.m.; **: *p* < 0.01, signed-rank test). (**I**) Grand mean slope of linear fits to gamma-centered Vm averages outside (Stim Off, black) and during (Stim On, grey) visual stimulation (*n* = 10; error bars: s.e.m.).

To understand how stimulation modulates gamma phase locking of PYRs, we repeated our analysis of Vm in our sample of pyramidal cells. As for PVs, gamma-centered Vm averages displayed a descending slope for weak gamma quintiles and an ascending slope as well as a clear depolarizing bump in strong quintiles (Fig 6B, 6E, 6H and 6I). Interestingly, visual stimulation resulted in a noticeable but non-significant increase of the average DC depolarization during strong gamma bouts (Fig 6G). During baseline and stimulation, coherence in the gamma range remained low in the weak gamma quintiles and increased markedly in the strongest quintile (Fig 6C and 6F). However, this increase reached significance exclusively during visual stimulation (Fig 6F). Thus, our results suggest that visual stimulation modulates gamma spike phase locking in PYRs through a combined mechanism whereby DC depolarization brings the cell closer to spike threshold and Vm oscillatory entrainment to gamma is strengthened.

## Discussion

Gamma band activity arises from the interplay of PVs and PYRs [20,21,30] and has been proposed to play an important role in cortical processing by synchronizing neurons within and across areas [2–4]. Nevertheless, while a wealth of theoretical models have described how rhythmic synchronization at gamma frequency can arise from networks of PVs and PYRs [22–24], the constraints applying to a realistic theoretical description of the temporal patterning of gamma activity and the entrainment of cortical neurons to gamma in the awake states have remained unclear.

In this study, we characterized the intracellular correlates of spontaneous and visually evoked gamma activity in the gamma rhythmogenic circuit of layers 2/3 of V1 of awake mice. The membrane potential dynamics of PVs and PYRS were recorded while monitoring spontaneous and visually evoked gamma oscillations in the LFP, giving us insights into the underlying dynamics of these oscillatory patterns. Gamma amplitude was divided over time into five quintiles, which allowed us to estimate the average behavior of PVs and PYRs while following the stochastic fluctuations of gamma phase and amplitude. Gamma amplitude remained moderate in the four first quintiles which together accounted for 80% of the recording time. However it increased dramatically in the strongest quintile. Epochs spent in the range of the strongest gamma quintile were typically short, lasting approximately one to three cycles (Fig 2E and 2F). These strong gamma bouts were evoked by visual stimulation but also occurred spontaneously (Fig 2C and 2D). While we found that the occurrence of strong gamma events increased during visual stimulation the distribution of their duration remained similar (Fig 2E and 2F). Thus, our data are consistent with the idea that awake gamma activity does not act as a global time reference but rather occurs as short synchronizing bouts [19,25]. We next investigated how the membrane potential dynamics of PVs and PYRs behaved as a function of gamma activity. Gamma power was positively correlated with depolarization in PVs and PYRs on a coarse (Fig 1F) and fine time scale (Figs 4I and 6I). In addition, we found that strong gamma bouts entrained coherent Vm fluctuations in PVs and PYRs both during baseline and visual stimulation (Figs 4B, 4E, 6B and 6E). Thus our data indicate that strong gamma bouts emerge when PVs and PYRs synchronize their membrane potential dynamics in the gamma range [31].

This important observation provides a potential explanation for the dynamics of LFP gamma activity in the awake state. In each cortical neuron, Vm is driven by thousands of excitatory and inhibitory synapses [46]. However most of this input is weak and arises from highly divergent local projections [46,47]. As a result, PVs and PYRs are driven to a large extent by a background synaptic input which reflects the activation of the neocortical network as a whole [43–45] and their membrane potential dynamics are highly correlated [41]. Our results thus indicate that the temporal patterning of gamma activity is a direct reflection of the dynamics of background synaptic activity [29,30]. During anesthesia, slow-wave sleep and to a lesser extent during quiet wakefulness, background synaptic activity is characterized by a slow alternation of depolarizing and hyperpolarizing states [44,48]. Gamma rides on the top of depolarizing states [30] which is consistent with observations suggesting that gamma strength is modulated by the phase of slower oscillations, a phenomenon known as cross-frequency coupling [49]. In turn, background synaptic activity becomes more sustained during visual stimulation and locomotion [35] which is consistent with the average increase in gamma power observed in these conditions [9,31,50]. Here, our finding suggests an explanation for the apparent random fluctuation of LFP gamma amplitude in awake states [25,26] whereby gamma strength is shaped by the stochastic dynamics of background synaptic activity on a fine time scale [51].

The stochastic temporal patterning of gamma activity raises the question of its functional relevance. Indeed, PV interneurons have been linked to a variety of cortical functions with no clear relationship to gamma synchronization such as feedforward inhibition, balancing of excitation, and gain control [36,52–54]. This has led some authors to propose that gamma is an epiphenomenon of the inhibitory function of PVs [6]. However, numerous studies have reported that excitatory neurons become entrained to gamma rhythmicity during selective behavioral epochs [8,9,11,12,55], and theoretical and experimental studies indicate that this enhances local processing as well as the impact of neuronal assemblies on higher order cortical areas [13–17,19]. Consistent with these findings, we found that the output of pyramidal cells phase locks to gamma during stimulation but not to gamma recorded in the absence of visual input. This implies that layers 2/3 can generate gamma activity in the absence of output from these layers to other areas, confirming the local nature of gamma rhythmogenesis under baseline or "idling" conditions. In addition, our results suggest a 2-fold mechanism for this selectivity. First, the average DC input to PYRs tends to be more depolarizing during visual stimulation (Fig 6G) thus bringing Vm closer to spike threshold. Second, the oscillatory power of Vm tends to be stronger (Fig 6H), which should facilitate action potential threshold crossing [56,57].

Where then does the synaptic input underlying gamma activity in PVs and PYRs of layer 2/3 come from? On a subset of our sample of PVs, we performed voltage clamp (VC) experiments in an attempt to disentangle the contribution of inhibitory and excitatory synaptic currents to Vm entrainment during strong gamma bouts (S4 Fig). VC recordings should be interpreted with caution [58]. Nevertheless, they suggest that excitatory currents, at least, play an important role in the entrainment of PVs at gamma during and outside visual stimulation, while not excluding an additional function of inhibitory currents. Layer 2/3 PVs and PYRs receive the majority of the excitatory drive from layer 2/3 and layer 4 [36,46,47,59,60], which leaves us with two most likely possibilities for the origin of these excitatory currents. In this study, layer 2/3 PYRs fired at very low rates and only became entrained to gamma activity during visual stimulation (Fig 5). Thus even though it remains possible that gamma locked excitatory inputs arise from a subset of very active layer 2/3 units [61] our data suggest that layer 2/3 PYRs are unlikely to be the sole provider of the excitatory drive during strong gamma bouts. Gamma might also propagate to layer 2/3 via the excitatory connections provided by layer 4. In accordance with this hypothesis, a recent in vivo study has found that gamma activity in layer 4 has a causal influence over the activity of layer 2/3 [15]. In addition, layer 4 immediately precedes the activation of layer 2/3 during spontaneous and evoked burst of background synaptic activity [62–64].

To our knowledge, this study represents the first characterization of the dynamics of the gamma rhythmogenic circuit in the awake state. Our results place constraints on possible theoretical models aiming at describing naturalistic gamma activity. In particular, they indicate that spontaneous and visually evoked gamma activity (1) is tightly linked to the dynamics of the background synaptic input underlying the membrane potential fluctuations of PVs and PYRs, (2) entrains the firing of PVs unconditionally (i.e., regardless of visual stimulation being on or off) and (3) entrains the firing of PYRs selectively during visual stimulation. The spectral characteristics of gamma activity described here are consistent with other studies performed in mouse [40,50] indicating that our results are representative in this species. However, our findings display some notable differences with results obtained in other model organisms. In particular, while visual stimulation results in a moderate and broad band increase of LFP power at gamma frequency in our conditions (Fig 1B), gamma-evoked activity is much more pronounced and narrow-banded in V1 of cat and monkeys [8,9,12,25,26]. On the other hand, the PPC spectra reported here show consistency with results obtained in monkey area V4 [55], raising the interesting possibility that complex visual processing could be subserved at the level of V1 in the mouse.

## Materials and Methods

### Transgenic Mice

All animal experiments were conducted after approval by the ethical committee (DEC) of the University of Amsterdam (Protocol number: DED235) in accordance with the Dutch “Experiment on Animal Act” and the European directive 86/609/EEC on the protection of animals used for experimental and other scientific purposes. We used heterozygous offspring of PV-ires-Cre driver mice (008069, Jackson) crossed with Ai9 loxP-tdTomato reporter mice (007909, Jackson) where red fluorescence is found selectively in parvalbumin-expressing neurons. Recordings of pyramidal neurons were performed with the shadow patching method on the same offsprings (*n* = 6) or in some instances, on offsprings of Ai9 and VIP-ires-Cre (010908, Jackson; *n* = 1) or SOM-ires-Cre (013044, Jackson; *n* = 4) mice. All animals used in this study were maintained on a C57Bl6 genetic background and group-housed in the vivarium under normal light cycle conditions.

### Awake Head Restrained Two-Photon-Targeted Patch-Clamp Recordings

Animals (6–12 wk old) were implanted with a lightweight head-bar and habituated to remain head-restrained during ~1 h for at least 5 d while being given regular sweet water rewards. On the day of recording, mice were anesthetized with ~2% isoflurane and the primary visual cortex (V1) was located on the skull using intrinsic optical imaging. A small craniotomy (1.5–2 mm) was performed above V1 and stabilized with a coverslip and 1.5% agar while leaving an opening on one side for pipette insertion. Animals were allowed to recover from anesthesia for at least 2 h before recording, and recording sessions lasted up to 4 h. Animals were placed on the stage of a Sutter MOM two-photon microscope combined with a pulsed Ti-Sapphire Mai-Tai Deep See Spectraphysics laser. Image and data acquisition was performed using the ScanImage and Ephus softwares (Janelia Farms). Simultaneous LFP and whole-cell/cell-attached recordings were performed using glass micropipettes filled respectively with ACSF (in mM: 135 NaCl, 5 KCl, 5 HEPES, 1 MgCl_2_, 1.8 CaCl_2_, 0.01 Alexa-488 [adjusted to pH 7.3 with NaOH]) and internal solution (in mM: 135 potassium gluconate, 4 KCl, 10 HEPES, 10 phosphocreatine, 4 MgATP, 0.3 Na_3_GTP, 0.01 Alexa-488 [adjusted to pH 7.3 with KOH; osmolarity adjusted to 300 mOsmol]). Unless otherwise noted, no current was injected during current clamp recording. On a subset of current clamp recordings, Vm was maintained under spike initiation threshold by negative current injections. Vm was not corrected for liquid junction potentials (Vj). Vj was, nevertheless, estimated as described previously [65] using extracellular ion concentration measured in vivo [66] (in mM: 153.5 Na^+^, 4.3 K^+^, 139.4 Cl^-^, 0.4 Mg^2+^, 0.7 Ca^2+^). The estimated Vj would bias Vm positively by 14.9 mV. Voltage clamp recordings were performed either at -80 mV or 10 mV to favor transmembrane currents respectively toward EPSCs or IPSCs (Vj corrected reversal potentials were estimated at -78 mV for K^+^, -80 mV for Cl^-^ and 18.1 mV for Na^+^/K^+^). Signals were acquired at 20 kHz and low passed Bessel filtered at 4 kHz with a Multiclamp 700 B amplifier (Molecular Devices). Recordings lasted on average 40 min for both whole-cell and cell-attached recordings (range 19 to 88 min). Sweet water rewards were delivered to the animal between recordings and its front paws hung onto a horizontal bar positioned in front of the animal in order to maintain wakefulness.

### Visual Stimulation

Visual stimuli were generated using the psychtoolbox Matlab extension and displayed on a small LCD screen (19 x 12.5 cm) placed ~11 cm in front of the eye of the animal in the contralateral hemifield. Stimulation appeared on a grey isoluminant background and consisted of sinusoidal drifting gratings (Spatial frequency: 0.04 cycles/degree, Temporal frequency: 1 cycles/s) displayed at full contrast on a circular area (radius: 15 degree) centered on the receptive field of recorded neurons. Gratings were displayed at one of eight possible directions for a duration 3 s, starting 2 s after the onset of 7 s long trials. A total of 40 to 80 trials were acquired for each recording (5–10 stimulus set repetitions). Grating direction was randomized across each stimulus set repetition.

### Data Preprocessing

Data processing and analyses were performed offline in the Matlab environment (Mathworks). Signals were notch-filtered at 50 Hz in order to remove spurious line noise. LFP signals were de-trended by subtracting a linear fit to traces on individual trials, low-pass filtered at 200 Hz and expressed as z-scores. Cell-attached recordings were high-passed filtered at 100 Hz. Spikes were detected on whole-cell and cell-attached recordings, using a threshold based procedure. The time corresponding to action potential threshold was taken as the first peak of the third derivative of the trace. For the analysis of membrane potentials (Vm), spikes were removed by interpolating trace segments from -1 ms to +3 ms around AP peaks with cubic splines.

### Orientation Selectivity

The orientation selectivity of recorded neurons was estimated with a standard Orientation Selectivity Index (OSI) defined as: *OSI* = (*R*_*pref*_—*R*_*orth*_)/(*R*_*pref*_ + *R*_*orth*_) where *R*_*pref*_ and *R*_*orth*_ respectively represent the mean firing rate during the presentation of gratings at the preferred direction and during the presentation of gratings at the orthogonal directions.

### Spectral Analysis

In order to compute LFP power spectra, LFP traces were divided into overlapping 500 ms segments spaced every 62.5 ms (16 segments per second). Each segment was multiplied by a Hamming taper and its Fourier transform was computed with the Fast Fourier Transform algorithm. The power spectral density of individual segments was computed as the squared modulus of the elements of the Fourier series divided by segment size and sample rate. Power spectra were derived by averaging power spectral densities over segments. Power (*P*) was expressed in decibel (10.log_10_(*P*)).

In order to track the instantaneous phase and amplitude of gamma activity on a fine time scale, we computed the so-called “analytic signal” of LFP traces band-pass filtered between 30 and 80 Hz [67]. The analytic signal is a complex valued representation where the real part corresponds to the signal itself and the imaginary part is given by the Hilbert transform of the signal. The instantaneous phase and amplitude of gamma were computed respectively as the complex argument (or angle) and the modulus of the analytic signal. In order to subdivide recordings into epochs as a function of gamma strength, the distribution of gamma amplitude over time was computed for each recording and divided in five quintiles. By definition, each quintile thus represents a range of gamma amplitudes within which one fifth of the total recording was spent. To estimate the duration of the epochs spent in the highest gamma quintile (i.e., the gamma bouts), we first detected the inflection points (peaks and valley) of the gamma-filtered LFP to define gamma hemicycles. Then the number of complete hemicycles contained in each epoch was counted and divided by 2 to express their duration in units of gamma cycles. For each recording, average firing rates were computed in each quintile by dividing the total number of spikes by recording time. For whole cell recordings, average direct current (DC) depolarization was computed in each quintile as the mean value of Vm.

To compute spike autocorrelograms, we first constructed “spike traces” having a value of one in windows of 1 ms centered on each spike and zero elsewhere. Spikes were then sorted per quintile as a function of the value of gamma amplitude at AP peak time. Autocorrelograms were computed for each quintile by averaging 150 ms segments of the above described “spike traces” centered on each spike. Spike-triggered averages (STAs) were computed similarly by averaging 150 ms spike-centered segments of the gamma-filtered LFP. STA amplitudes were derived with a Hilbert transform without additional filtering and the oscillatory power of STAs was computed as the sum of the squared values of STA amplitude divided by segment duration. Gamma centered Vm averages were derived by first sorting gamma cycles per quintile as a function of gamma amplitude at cycle trough. Then, 50 ms Vm segments centered on the trough of each cycle were aligned and averaged. A linear fit was computed on each 50 ms Vm average to derive its slope. Then, this fit was subtracted from the Vm average and the amplitude of the gamma entrained Vm fluctuation was calculated as the maximum of the resulting trace.

For the assessment of the phase locking of spike and Vm to the LFP, a continuous spectro-temporal representation of LFPs and membrane potentials was derived from 2 to 120 Hz (step 2 Hz) with a Continuous Wavelet Transform using a Complex Morlet Wavelet having 9 cycles (bandwidth parameter: 1, center frequency: 2, wavelet name: “cmor1-2” in the Matlab Wavelet toolbox).

The strength of spike-LFP phase locking was quantified using the Pairwise Phase Consistency (PPC) [68,69]. PPC is unbiased by the total number of spikes and is defined for a given frequency *f* as:

$$\hat{PPC_{f}}\;=\;\frac{\sum_{m\;=\;1}^{M}\sum_{l\neq m}^{M}\sum_{j\;=\;1}^{N_{m}}\sum_{k\;=\;1}^{N_{l}}\text{cos}(\theta_{l,k}-\theta_{m,j})}{\sum_{m\;=\;1}^{M}\sum_{l\neq m}^{M}N_{m}N_{l}}$$

where *m* and *l* represent the *m*-th and *l*-th trial out of *M* total trials, *j* is the *j*-th spike of trials *m* and *k* the *k*-th spike of trial *l*, *N*_*m*_ and *N*_*l*_ are the total number of spikes of trials *m* and *l* respectively and *θ*_*m*,*j*_ represents the phase of the spectro-temporal temporal representation of the LFP at the time of the *j*-th spike of trial *m*. PPC provides a reliable estimate of phase locking when the total number of spikes in a recording is roughly over 250 (S2 and S6 Figs). This condition was largely fulfilled for recording of PVs. Thus PPC was calculated for every recording and averaged over recordings. On the other hand, PYR recordings often contained a low number of spikes and yielded noisy PPC estimates on single recordings. In order to circumvent this problem, PYR recordings were pooled and PPC was calculated over our complete pyramidal cell sample.

The strength of phase locking between LFP and membrane potential was quantified using the squared coherence [67] and was estimated for a given frequency *f* as:

$$\hat{\kappa_{f}}\;=\;\frac{{\left|\sum_{n\;=\;1}^{N}S_{Vm}\left(n\right).S_{LFP}^{\text{*}}\left(n\right)\right|}^{2}}{\sum_{n\;=\;1}^{N}{\left|S_{Vm}\left(n\right)\right|}^{2}.\sum_{n\;=\;1}^{N}{\left|S_{LFP}\left(n\right)\right|}^{2}}$$

Where *S*_*x*_(*n*) is the complex valued spectro-temporal representation of signal *X* at time point *n* of *N* total time points and * denotes complex conjugation. This estimator has a positive bias of $(1-\hat{\kappa_{f}})/N$ which was subtracted from $\hat{\kappa_{f}}$ for correction.

### Statistics

Otherwise noted, statistical comparisons between paired and unpaired observations were performed respectively with Wilcoxon signed rank tests and Mann-Whitney U tests. For spectra, multiple comparisons were corrected with a Benjamini-Hochberg-Yekutieli False Detection Rate procedure (FDR) [70]. For PPC on PYRs, 95% confidence intervals were computed with a leave one out Jackknife approach [71]. Briefly, the variance of the estimate was estimated as $\hat{V}\;=\;\frac{n-1}{n}\sum_{i\;=\;1}^{n}{\left(\hat{{PPC}_{i}}-\hat{PPC}\right)}^{2}$, where *n* is the number of neurons included, $\hat{{PPC}_{i}}$ represents the PPC estimate when the contribution of the *i*-th cell is omitted and $\hat{PPC}$ represents the estimate of PPC when all neurons are included. Then, 95% confidence intervals where computed using the percentiles of a normal distribution of variance $\hat{V}$. PPC values were considered significantly different if there was no overlap between their 95% confidence intervals. Non-uniformities in the phase distribution of spikes were tested with Rayleigh’s tests. As Rayleigh’s test tends to be permissive, the threshold for significance was set to α = 10^−5^. All values are presented as mean ± s.e.m. unless otherwise stated. Data deposited in the Dryad repository: http://dx.doi.org/10.5061/dryad.4754j [72].

## Supporting Information

S1 Fig Gamma phase locking does not affect the fine-scale rhythmic properties of spike trains in PVs. (**A, C**) Grand mean spike autocorrelograms of PVs, outside (A) and during (C) visual stimulation, as a function of gamma quintile at spike time (*n* = 23; shaded areas: +/- s.e.m.). (**B, D**) Grand mean PV Spike Triggered Averages (STAs) of gamma-filtered LFPs, outside (B) and during (D) visual stimulation, as a function of gamma quintile at spike time (*n* = 23; shaded areas: +/- s.e.m.). (**E**) Grand mean oscillatory power of PV STAs outside (Stim Off, black) and during (Stim On, grey) visual stimulation in a window of 50 ms around spike time (*n* = 23; error bars: s.e.m.; **: *p* < 0.01, signed rank test). (**F**) Spike threshold remains unchanged across gamma quintiles for PVs (*n* = 10; error bars: s.e.m.; *p* = 0.9969; Kruskal-Wallis one-way ANOVA).
(PNG)

S2 Fig PPC in PVs as a function of spike sample size. (**A-H**) Average Spike-LFP Pairwise Phase Consistency (PPC) estimates over 1,000 independent resamplings outside (A–D) and during (E–H) visual stimulation. Fixed numbers of spikes per cell and per condition where used (A, E: 125 spikes; B, F; 250 spikes; C, G: 500 spikes; D, H: 1,000 spikes; *n* = 23 cells) and estimates and statistical significances were computed as in Fig 3C and 3F. Using larger spike samples increases the reliability of the estimates but has little effect on their average values (light and dark blue traces: average PPC estimates respectively in the four weakest quintiles and in the strongest gamma quintile; shaded areas: interval containing 95% of the estimates; horizontal lines: proportion of statistically significant differences between the four weakest quintiles and the strongest gamma quintile (black) and between Stim Off and Stim On for the four weakest quintiles (light blue) and the strongest gamma quintile (dark blue), FDR corrected signed-rank test, α = 0.05).
(PNG)

S3 Fig Strong gamma oscillations entrain subthreshold Vm in PVs. (**A**) Example trace of a recording of a PV interneuron where current was injected to maintain Vm subthreshold. The resting Vm was held around -80 mV (top: whole-cell recording; middle: gamma-filtered LFP (light blue) and gamma amplitude envelope computed with the Hilbert transform (dark blue); bottom: gamma quintiles color coded as in (C); grey rectangle: visual stimulation period). (**B**) Enlargement of the portion enclosed in the black rectangle in (A) showing examples of spontaneous gamma bouts. (**C, F**) Grand mean of trough-centered segments of the gamma-filtered LFP, outside (C) and during (F) visual stimulation, as a function of gamma quintile at trough time (*n* = 6; shaded areas: +/- s.e.m.). (**D, G**) Grand mean of simultaneously recorded PV subthreshold Vm segments (*n* = 6; shaded areas: +/- s.e.m.). (**E, H**) Strong gamma oscillations increase PV subthreshold Vm-LFP coherence in the gamma range (30–80 Hz) outside (E) and during (H) visual stimulation (*n* = 6; light and dark blue traces: grand mean coherence respectively in the four weakest quintiles and in the strongest gamma quintile; shaded areas: +/- s.e.m; no statistical difference was observed between the four weakest quintiles and the strongest gamma quintile after FDR correction, FDR corrected signed-rank test, α = 0.05). (**I**) Grand mean subthreshold DC Vm of PVs as a function of gamma quintile outside (Stim Off, black) and during (Stim On, grey) visual stimulation (*n* = 6; error bars: s.e.m.; *: *p* < 0.05, signed-rank test). (**J**) Grand mean amplitude of subthreshold gamma-centered Vm averages outside (Stim Off, black) and during (Stim On, grey) visual stimulation (*n* = 6; error bars: s.e.m.; *: *p* < 0.05, signed-rank test). (**K**) Grand mean slope of linear fits to subthreshold gamma-centered Vm averages outside (Stim Off, black) and during (Stim On, grey) visual stimulation (*n* = 6; error bars: s.e.m.).
(PNG)

S4 Fig EPSCs contribute to Gamma in PV interneurons. (**A, B**) Example Voltage Clamp recordings of PV dominated either by EPSCs (A: holding potential -80 mV) or IPSCs (B: holding potential: +10 mV; top: whole-cell recording; middle: gamma-filtered LFP (light blue) and gamma amplitude envelope computed with the Hilbert transform (dark blue); bottom: gamma quintiles color coded as in (C); grey rectangle: visual stimulation period). (**A, G, E, F**) Grand mean of trough-centered segments of the gamma-filtered LFP, outside (C, E) and during (G, I) visual stimulation, as a function of gamma quintile at trough time (*n* = 4; shaded areas: +/- s.e.m.). (**D, F, H, J**) Grand mean gamma trough centered transmembrane currents segments recorded at holding potential: -80 mV (D, H) or +10 mV (F, J) in PVs (*n* = 4; shaded areas: +/- s.e.m.). (**K, L, M, N**) The coherence in the gamma range (30–80 Hz) tends to be stronger for EPSC- (K, M) and IPSC-dominated traces (L, N) in strong gamma quintiles, outside (K, L) and during (M, N) visual stimulation (*n* = 4; shaded areas: +/- s.e.m; No statistical difference was observed between conditions, FDR corrected paired *t* test, α = 0.05). It should be noted that the less pronounced IPSC averages may relate to higher noise levels, contamination by other ionic currents and/or incomplete voltage clamp at +10 mV.
(PNG)

S5 Fig Gamma phase locking does not affect the fine-scale rhythmic properties of spike trains in PYRs. (**A, C**) Grand mean spike autocorrelograms of PYRs, outside (A) and during (C) visual stimulation, as a function of gamma quintile at spike time (*n* = 10; shaded areas: +/- s.e.m.). (**B, D**) Grand mean PYR STAs of gamma-filtered LFPs, outside (B) and during (D) visual stimulation, as a function of gamma quintile at spike time (*n* = 10; shaded areas: +/- s.e.m.). (**E**) Grand mean oscillatory power of STAs outside (Stim Off, black) and during (Stim On, grey) visual stimulation in a window of 50 ms around spike time (*n* = 10; error bars: s.e.m.; no significant differences were observed, signed rank test).
(PNG)

S6 Fig PPC in PYRs as a function of spike sample size. (**A–H**) Average pooled Spike-LFP Pairwise Phase Consistency (PPC) estimates over 1,000 independent resamplings outside (A–D) and during (E–H) visual stimulation. Fixed numbers of spikes per condition where used (A, E: 125 spikes; B, F; 250 spikes; C, G: 500 spikes; D, H: 1,000 spikes; pooled from *n* = 10 cells) and estimates and statistical significances were computed as in Fig 5C and 5F.
(PNG)

## Abbreviations

EPSC: excitatory post synaptic current
FDR: Benjamini-Hochberg-Yekutieli False Detection Rate correction procedure for multiple comparisons
IPSC: inhibitory post synaptic current
LFP: Local Field Potential
OSI: Orientation Selectivity Index
PPC: pairwise phase consistency
PV: parvalbumin-expressing interneuron
PYR: excitatory pyramidal neuron
SD: standard deviation
s.e.m.: standard error of the mean
STA: spike-triggered average
Stim Off: period of visual presentation of a grey background
Stim On: period of visual presentation of drifting gratings
TPTP: two-photon targeted patch-clamp recordings
V1: primary visual cortex
VC: voltage clamp
Vm: membrane potential
WC: whole-cell
