# Supplementary figures and images for: Membrane Potential Dynamics of Spontaneous and Visually Evoked Gamma Activity in V1 of Awake Mice
Source: PLoS Biol. 2016 Feb 18;14(2):e1002383. doi: 10.1371/journal.pbio.1002383 (PMC4758619; doi:10.1371/journal.pbio.1002383)

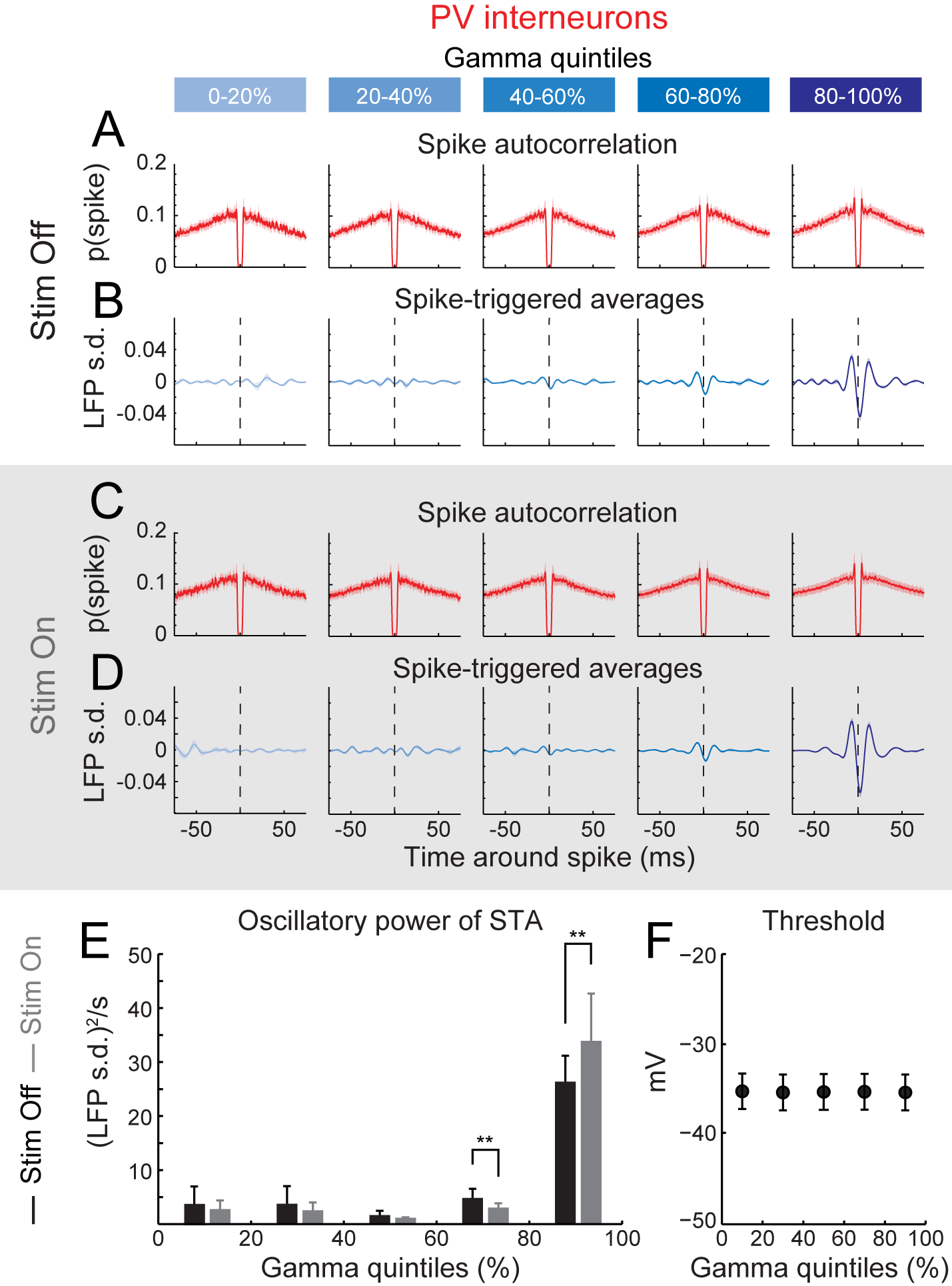

Supplement: S1 Fig — (A, C) Grand mean spike autocorrelograms of PVs, outside (A) and during (C) visual stimulation, as a function of gamma quintile at spike time (n = 23; shaded areas: +/- s.e.m.). (B, D) Grand mean PV Spike Triggered Averages (STAs) of gamma-filtered LFPs, outside (B) and during (D) visual stimulation, as a function of gamma quintile at spike time (n = 23; shaded areas: +/- s.e.m.). (E) Grand mean oscillatory power of PV STAs outside (Stim Off, black) and during (Stim On, grey) visual stimulation in a window of 50 ms around spike time (n = 23; error bars: s.e.m.; **: p < 0.01, signed rank test). (F) Spike threshold remains unchanged across gamma quintiles for PVs (n = 10; error bars: s.e.m.; p = 0.9969; Kruskal-Wallis one-way ANOVA). (PNG) [file pbio.1002383.s001.png]

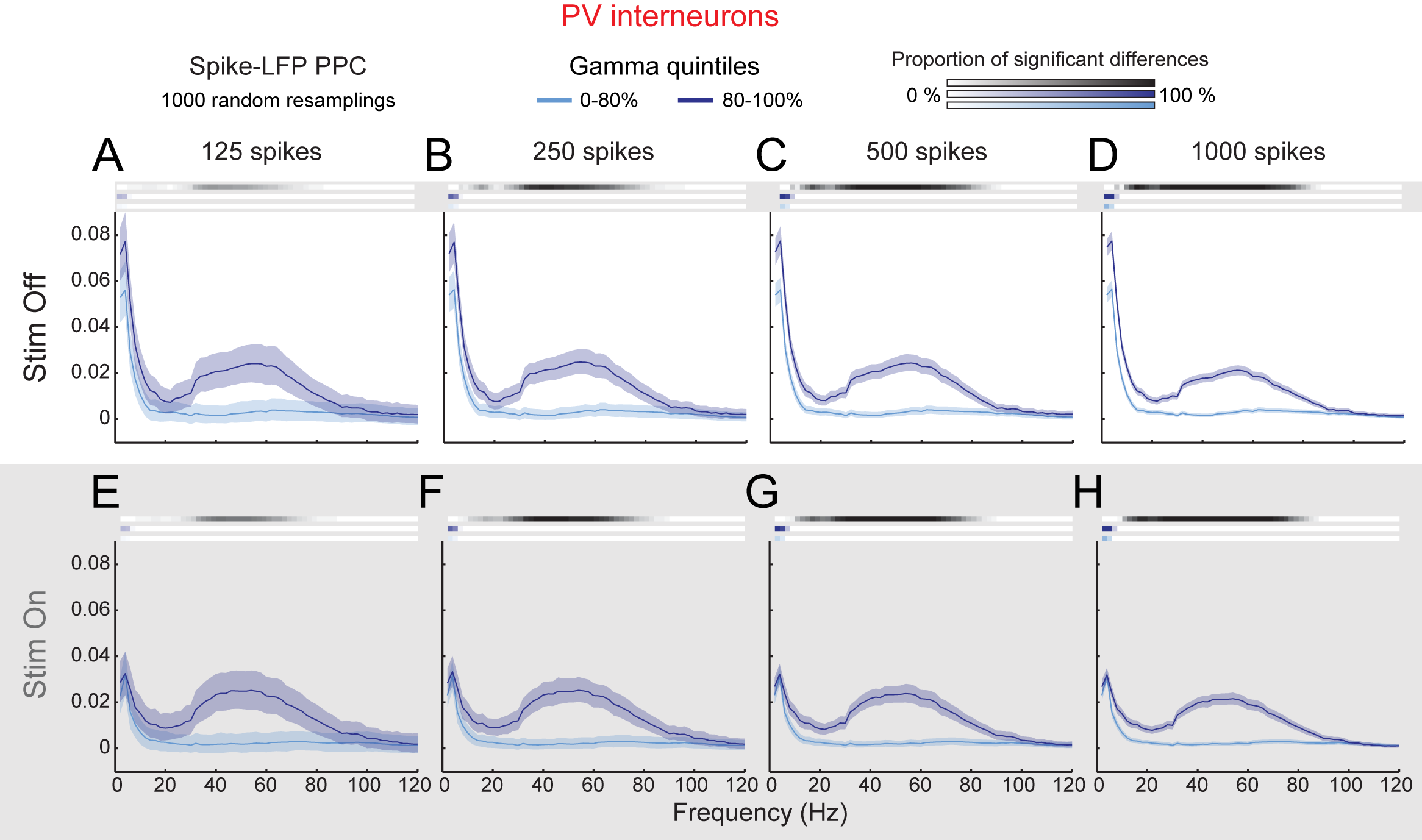

Supplement: S2 Fig — (A-H) Average Spike-LFP Pairwise Phase Consistency (PPC) estimates over 1,000 independent resamplings outside (A–D) and during (E–H) visual stimulation. Fixed numbers of spikes per cell and per condition where used (A, E: 125 spikes; B, F; 250 spikes; C, G: 500 spikes; D, H: 1,000 spikes; n = 23 cells) and estimates and statistical significances were computed as in Fig 3C and 3F. Using larger spike samples increases the reliability of the estimates but has little effect on their average values (light and dark blue traces: average PPC estimates respectively in the four weakest quintiles and in the strongest gamma quintile; shaded areas: interval containing 95% of the estimates; horizontal lines: proportion of statistically significant differences between the four weakest quintiles and the strongest gamma quintile (black) and between Stim Off and Stim On for the four weakest quintiles (light blue) and the strongest gamma quintile (dark blue), FDR corrected signed-rank test, α = 0.05). (PNG) [file pbio.1002383.s002.png]

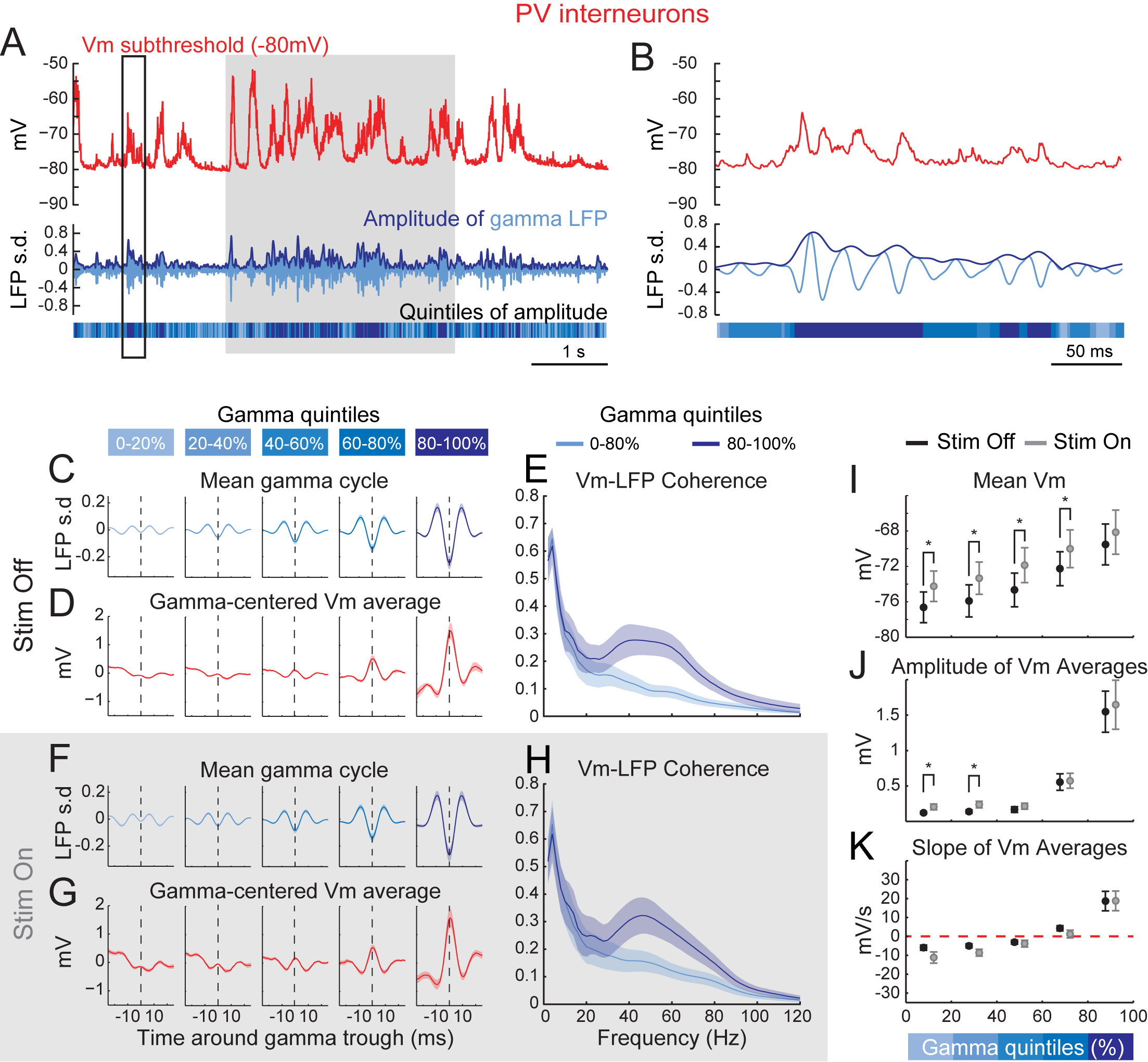

Supplement: S3 Fig — (A) Example trace of a recording of a PV interneuron where current was injected to maintain Vm subthreshold. The resting Vm was held around -80 mV (top: whole-cell recording; middle: gamma-filtered LFP (light blue) and gamma amplitude envelope computed with the Hilbert transform (dark blue); bottom: gamma quintiles color coded as in (C); grey rectangle: visual stimulation period). (B) Enlargement of the portion enclosed in the black rectangle in (A) showing examples of spontaneous gamma bouts. (C, F) Grand mean of trough-centered segments of the gamma-filtered LFP, outside (C) and during (F) visual stimulation, as a function of gamma quintile at trough time (n = 6; shaded areas: +/- s.e.m.). (D, G) Grand mean of simultaneously recorded PV subthreshold Vm segments (n = 6; shaded areas: +/- s.e.m.). (E, H) Strong gamma oscillations increase PV subthreshold Vm-LFP coherence in the gamma range (30–80 Hz) outside (E) and during (H) visual stimulation (n = 6; light and dark blue traces: grand mean coherence respectively in the four weakest quintiles and in the strongest gamma quintile; shaded areas: +/- s.e.m; no statistical difference was observed between the four weakest quintiles and the strongest gamma quintile after FDR correction, FDR corrected signed-rank test, α = 0.05). (I) Grand mean subthreshold DC Vm of PVs as a function of gamma quintile outside (Stim Off, black) and during (Stim On, grey) visual stimulation (n = 6; error bars: s.e.m.; *: p < 0.05, signed-rank test). (J) Grand mean amplitude of subthreshold gamma-centered Vm averages outside (Stim Off, black) and during (Stim On, grey) visual stimulation (n = 6; error bars: s.e.m.; *: p < 0.05, signed-rank test). (K) Grand mean slope of linear fits to subthreshold gamma-centered Vm averages outside (Stim Off, black) and during (Stim On, grey) visual stimulation (n = 6; error bars: s.e.m.). (PNG) [file pbio.1002383.s003.png]

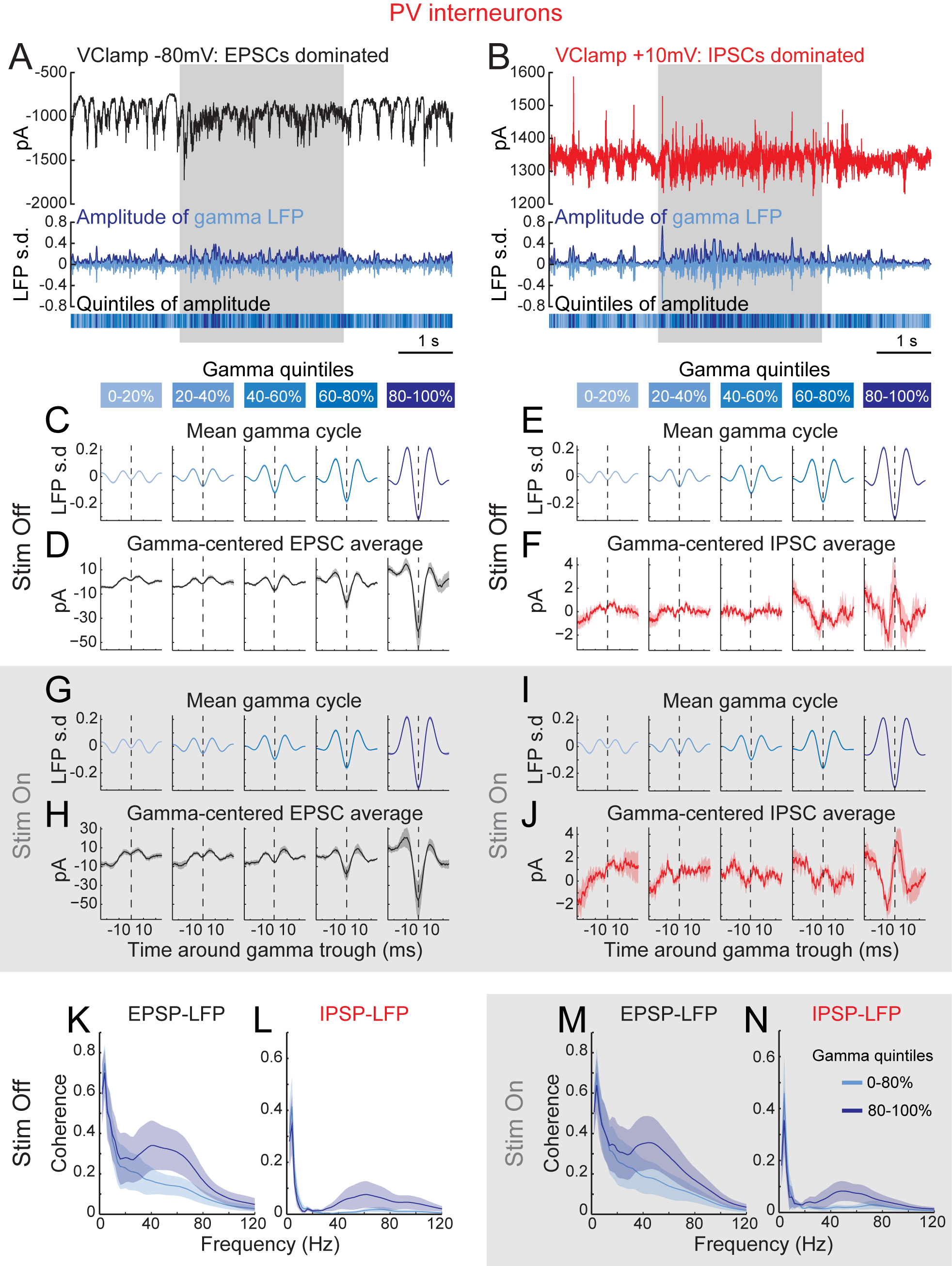

Supplement: S4 Fig — (A, B) Example Voltage Clamp recordings of PV dominated either by EPSCs (A: holding potential -80 mV) or IPSCs (B: holding potential: +10 mV; top: whole-cell recording; middle: gamma-filtered LFP (light blue) and gamma amplitude envelope computed with the Hilbert transform (dark blue); bottom: gamma quintiles color coded as in (C); grey rectangle: visual stimulation period). (A, G, E, F) Grand mean of trough-centered segments of the gamma-filtered LFP, outside (C, E) and during (G, I) visual stimulation, as a function of gamma quintile at trough time (n = 4; shaded areas: +/- s.e.m.). (D, F, H, J) Grand mean gamma trough centered transmembrane currents segments recorded at holding potential: -80 mV (D, H) or +10 mV (F, J) in PVs (n = 4; shaded areas: +/- s.e.m.). (K, L, M, N) The coherence in the gamma range (30–80 Hz) tends to be stronger for EPSC- (K, M) and IPSC-dominated traces (L, N) in strong gamma quintiles, outside (K, L) and during (M, N) visual stimulation (n = 4; shaded areas: +/- s.e.m; No statistical difference was observed between conditions, FDR corrected paired t test, α = 0.05). It should be noted that the less pronounced IPSC averages may relate to higher noise levels, contamination by other ionic currents and/or incomplete voltage clamp at +10 mV. (PNG) [file pbio.1002383.s004.png]

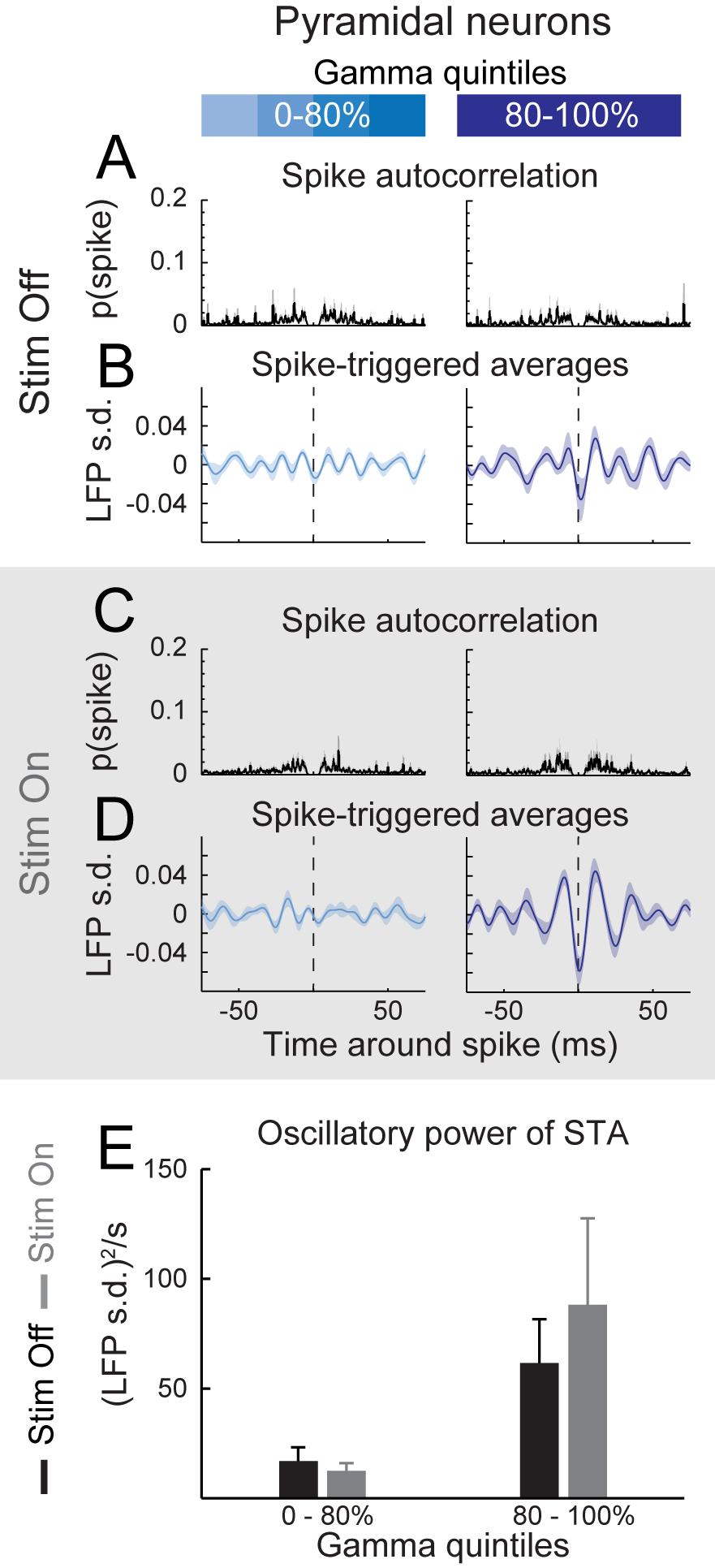

Supplement: S5 Fig — (A, C) Grand mean spike autocorrelograms of PYRs, outside (A) and during (C) visual stimulation, as a function of gamma quintile at spike time (n = 10; shaded areas: +/- s.e.m.). (B, D) Grand mean PYR STAs of gamma-filtered LFPs, outside (B) and during (D) visual stimulation, as a function of gamma quintile at spike time (n = 10; shaded areas: +/- s.e.m.). (E) Grand mean oscillatory power of STAs outside (Stim Off, black) and during (Stim On, grey) visual stimulation in a window of 50 ms around spike time (n = 10; error bars: s.e.m.; no significant differences were observed, signed rank test). (PNG) [file pbio.1002383.s005.png]

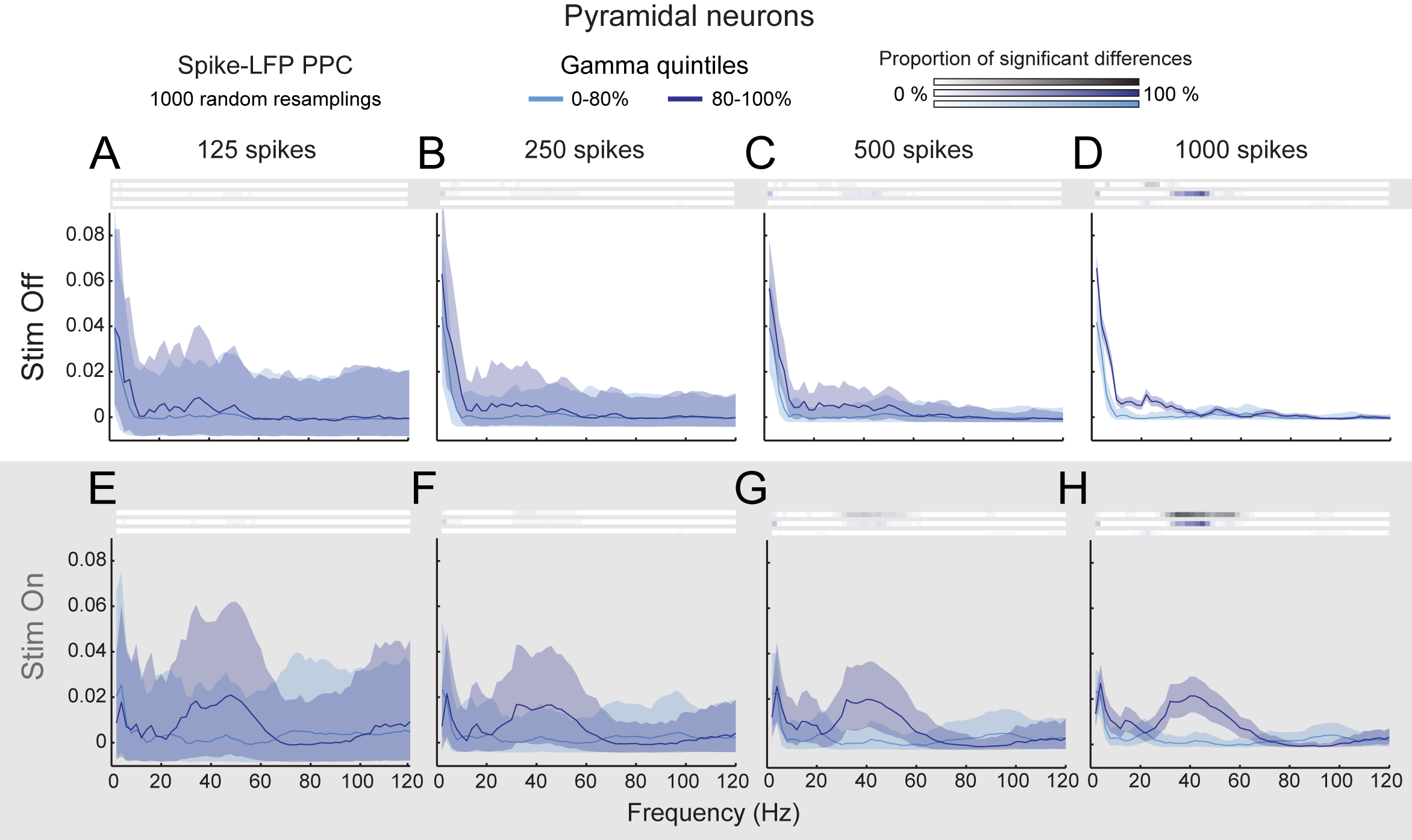

Supplement: S6 Fig — (A–H) Average pooled Spike-LFP Pairwise Phase Consistency (PPC) estimates over 1,000 independent resamplings outside (A–D) and during (E–H) visual stimulation. Fixed numbers of spikes per condition where used (A, E: 125 spikes; B, F; 250 spikes; C, G: 500 spikes; D, H: 1,000 spikes; pooled from n = 10 cells) and estimates and statistical significances were computed as in Fig 5C and 5F. (PNG) [file pbio.1002383.s006.png]
